# Region and layer-specific expression of GABA_A_ receptor isoforms and KCC2 in developing cortex

**DOI:** 10.3389/fncel.2024.1390742

**Published:** 2024-06-04

**Authors:** Kirill Zavalin, Anjana Hassan, Yueli Zhang, Zain Khera, Andre H. Lagrange

**Affiliations:** ^1^Department of Neurology, Vanderbilt University School of Medicine, Nashville, TN, United States; ^2^Department of Neurology, TVH VA Medical Center, Nashville, TN, United States

**Keywords:** GABA-A receptors, potassium chloride co-transporter 2 (KCC2), developmental expression pattern, cortical development, GABAA subtypes, Western blot (WB), immunohistochemistry (IHC)

## Abstract

**Introduction:**

γ-Aminobutyric acid (GABA) type A receptors (GABA_A_Rs) are ligand-gated Cl-channels that mediate the bulk of inhibitory neurotransmission in the mature CNS and are targets of many drugs. During cortical development, GABA_A_R-mediated signals are significantly modulated by changing subunit composition and expression of Cl-transporters as part of developmental processes and early network activity. To date, this developmental evolution has remained understudied, particularly at the level of cortical layer-specific changes. In this study, we characterized the expression of nine major GABA_A_R subunits and K-Cl transporter 2 (KCC2) in mouse somatosensory cortex from embryonic development to postweaning maturity.

**Methods:**

We evaluated expression of α1-5, β2-3, γ2, and δ GABA_A_R subunits using immunohistochemistry and Western blot techniques, and expression of KCC2 using immunohistochemistry in cortices from E13.5 to P25 mice.

**Results:**

We found that embryonic cortex expresses mainly α3, α5, β3, and γ2, while expression of α1, α2, α4, β2, δ, and KCC2 begins at later points in development; however, many patterns of nuanced expression can be found in specific lamina, cortical regions, and cells and structures.

**Discussion:**

While the general pattern of expression of each subunit and KCC2 is similar to previous studies, we found a number of unique temporal, regional, and laminar patterns that were previously unknown. These findings provide much needed knowledge of the intricate developmental evolution in GABA_A_R composition and KCC2 expression to accommodate developmental signals that transition to mature neurotransmission.

## Introduction

GABA-A receptors (GABA_A_Rs) are Cl^−^-conducting ion channels activated by γ-Aminobutyric acid (GABA) that convey the majority of inhibitory neurotransmission in the adult brain. GABA_A_R activity appears early in cortical development, preceding the emergence of glutamatergic activity in some brain regions (Wang and Kriegstein, 2008; Chancey et al., 2013; Warm et al., 2022). This early GABAergic signaling regulates many aspects of brain development, including migration of GABAergic interneuron progenitors (Cuzon et al., 2006; Bortone and Polleux, 2009; Cuzon Carlson and Yeh, 2011; Inada et al., 2011; Inamura et al., 2012), formation of synapses (Wang and Kriegstein, 2008, 2011; Oh et al., 2016), neurite extension (Ge et al., 2006; Cancedda et al., 2007; Bouzigues et al., 2010), and circuit integration of immature neurons through concerted high-amplitude early network oscillations termed “giant depolarizing potentials” (Ben-Ari et al., 2007; Allene et al., 2008); reviewed by Kilb (2021) and Peerboom and Wierenga (2021).

GABA_A_Rs are pentameric ligand-gated ion channels, commonly comprised of a combination of α, β, and either γ or δ subunits that determine receptor properties and localization. There are multiple isoforms for these subunits, with α1-5, β1-3, γ2, and δ being the most prevalent in mammalian forebrain. Of the most common subunit combinations, ɑ1β2/3γ2, ɑ2β2/3γ2, and ɑ3β2/3γ2 localize to synapses and mediate phasic responses to GABA, while ɑ4βδ and ɑ5βγ2 are typically found in the extrasynaptic space and primarily mediate tonic currents (Chuang and Reddy, 2018; Engin et al., 2018). The ɑ1β2/3γ2 combination is the predominant subunit combination found in mature synapses and the target of many of our most useful drugs used in babies and adults, including anti-seizure medicine and anxiolytics (Möhler, 2006; Engin et al., 2018). In contrast, α4β2δ and α5β3γ2 GABA_A_Rs that mediate tonic inhibition are sensitive to lower and [GABA] and have slower activation and deactivation kinetics (Lagrange et al., 2018). These receptors are targeted by several general anesthetics and alcohol. Many subunit isoforms have regionally specific distributions (Pirker et al., 2000; Hortnagl et al., 2013).

GABA_A_R-mediated responses undergo a significant transformation over the course of development that reflects synaptic maturation, but also transient developmental adaptations. Most notably, GABA_A_R activation during early development can be excitatory, which triggers Ca^2+^ transients that promote cytoskeletal remodeling and synaptic plasticity and appears to be essential for many developmental processes driven by GABA (Kilb, 2021; Peerboom and Wierenga, 2021). On a similar timeline, early GABAergic responses display slow and tonic kinetics conducive to developmental processes (Owens et al., 1999; Daw et al., 2007; Sebe et al., 2010; Le Magueresse and Monyer, 2013; Warm et al., 2022), while fast GABAergic responses optimal for resolution of discrete synaptic events emerge with maturity (Bosman et al., 2002; Kobayashi et al., 2008; Brown et al., 2016; Kroon et al., 2019).

Developmental changes in expression of GABA_A_R subunits and K-Cl transporter 2 (KCC2) drive many of the changes to GABAergic responses during development. Cl^−^ extrusion by KCC2 is the primary mechanism for maintaining low intracellular [Cl^−^] that drives inhibitory GABAergic responses. Both KCC2 expression and kinase-determined functional state are developmentally regulated to increase KCC2 activity with maturation of GABAergic neurotransmission, resulting in a relatively rapid shift from excitatory to inhibitory GABAergic responses in the second and third week of postnatal life (Fukuda, 2020). Meanwhile, a large body of work has demonstrated that developmental changes in subunit composition of GABA_A_Rs profoundly modify GABAergic signaling in context of specific developmental processes (Bosman et al., 2002; Serwanski et al., 2006; Giusi et al., 2009; Sebe et al., 2010; Cuzon Carlson and Yeh, 2011; Brady and Jacob, 2015; Hernandez et al., 2019; Lodge et al., 2021).

Given the importance of KCC2 and GABA_A_R composition during development, a detailed understanding of their developmental expression is of vital importance. Previously, several expression studies (Fritschy et al., 1994; Golshani et al., 1997; Paysan et al., 1997) characterized the general and regional course of GABA_A_R subunit expression in developing cortex, including an extensive characterization of mRNA expression of thirteen major GABA_A_R subunits from middle of embryonic development to adulthood in rat brains (Laurie et al., 1992). Unfortunately, these studies are limited by primarily looking only at mRNA expression and missing laminar details, and a need exists for a more comprehensive, detailed characterization of GABA_A_R subunit protein expression similar to adult expression studies (Pirker et al., 2000; Hortnagl et al., 2013). A lesser knowledge gap exists for developmental expression of KCC2, which has been investigated at the level of mRNA and protein in mouse and human tissue, including regional specificity in adult CNS (Markkanen et al., 2014) and developmental expression (Lu et al., 1999; Rivera et al., 1999; Stein et al., 2004; Uvarov et al., 2009; Murguia-Castillo et al., 2013), specifically including cortex (Dzhala et al., 2005; Vanhatalo et al., 2005; Takayama and Inoue, 2010; Kovács et al., 2014; Sedmak et al., 2016). These studies defined the general trend in KCC2 expression over the course of development, including some laminar and cell-type specificity, such as early interneuron-specific expression we recently reported (Zavalin et al., 2022). However, a more comprehensive study of KCC2 expression in developing cortical lamina is still needed. In this study, we address these knowledge gaps by a comprehensive and focused characterization of expression patterns of major GABA_A_R subunits and KCC2 from cortical plate formation (E13.5) to more mature brain (P26) in mouse cortex. We paid particular attention to lamina-specific expression within barrel cortex, which showed a rich level of complexity at these ages.

## Materials and methods

### Experimental mice, background and breeding

Mice were maintained in temperature-controlled housing areas, were adequately fed, hydrated, and kept under 12:12 h of alternating dark/light cycles. All animal handling and procedures were approved by Vanderbilt IACUC and VA ACORP committees. All experiments were performed using both female and male C57BL/6J congenic mice. Experiments requiring interneuron identification used Dlx5/6-Cre-IRES-EGFP mice (Jackson labs stock # 023724) that we had bred into the C57BL/6J congenic line for at least eleven generations. *Gabra3* knockout mice, exhibiting complete loss of GABA_A_R α3, were generously donated by Uwe Rudolph, maintained by our lab, and used for validating the GABA_A_R α3 antibody from Alomone labs. For experiments requiring embryonic timepoints, timed pregnancies for dams were carried out by mating them with wild type males from 4 pm to 4 pm next day. The following day in which the vaginal mucous plug was seen was taken as E0.5.

### Tissue collection and preparation

Postnatal brain tissues for P1, P5, P12, and P25/26 timepoints were collected from either wildtype or *Dlx*5*GFP*^+/WT^ mice to label MGE-derived interneurons by decapitation under isoflurane anesthesia, after which the brain and meninges were removed from the skull. Similarly, E13.5, E15.5, and E17.5 brain tissues were collected from embryos that were dissected from pregnant dams under anesthesia. After dissection, the embryos were quickly decapitated. For Western blot (WB) experiments, brains were dissected in PBS with tweezers under a dissection microscope to separate cortex from subcortical structures, and then processed as described below. For immunohistochemistry (IHC) experiments, embryonic heads and postnatal brain tissue were fixed with a brief immersion in 4% paraformaldehyde in PBS for 7 min. Similarly to previous reports (Schneider Gasser et al., 2006), we found that this light fixation protocol provided much greater detail for GABA_A_R studies than the relatively homogenous, non-punctate staining that we typically obtained from cardiac-perfused tissue. Following fixation, postnatal brains and embryonic heads were transferred to 30% sucrose for 24–48 h for cryoprotection, blocked by coronal cuts at the levels of prefrontal cortex and brain stem, and cryo-embedded in OCT compound. Twenty μm-thick sections were obtained for all ages under study using a Leica cryostat and stored in −80°C.

### Genotyping, PCR and primers

PCR analysis was performed on tail tissue harvested on E13.5, E15.5, E17.5, P1, P5, or on ear punches for P12 and P25 to determine genotypes. We used Sigma REDExtract-N-Amp tissue PCR kit Cat # XNAT-100RXN for extracting and amplifying the tissue DNA. For genotyping presence of Dlx5/6-Cre-IRES-EGFP in our mice, we used the following primers: Cre Forward 5′-GCA TTA CCG GTC GAT GCA ACG AGT GAT GAG-3′, Cre reverse: 5′-GAG TGA ACG AAC CTG GTC GAA ATC AGT GCG-3′ and the following thermal cycler protocol: 94°C for 3 min, (94°C for 30 seconds, 68°C for 30 seconds, 72°C for 1 min) × 30 cycles to amplify a cre product at 408 bp. For genotyping *Gabra3* knockout mice, we used the following primers: Primer UR75: 5′-GAC AGA CAT GGC ATG ATG AAA GAC TGA AAT−3′, Primer UR106: 5′-ACA AAA TGT AAG AAC AAG AAC CAA GAA AAT-3′ and the following thermal cycler protocol: 96°C for 1 min, (96°C for 15 seconds, 50°C for 10 seconds, 70°C for 1 min) × 40 cycles, 68°C for 5 min and hold at 4°C to amplify a single band at 480 bp for wildtype and two bands at 480 and 520 bp for knockout. Product bands were distinguished by electrophoresis on a 2% agarose gel stained with SYBR Safe DNA gel stain (Invitrogen Cat# P/N S33102) and visualized using Biorad GelDoc EZ.

### Antibodies

We chose target proteins and antibodies for this study based on several factors. Firstly, we reviewed known mRNA expression in embryonic/perinatal forebrain (Laurie et al., 1992). We then chose subunits contained in GABA_A_R combinations whose pharmacological and kinetic properties have been characterized to allow us to formulate subsequent hypotheses about the potential physiological significance of our results. Finally, we selected commercially available antibodies to improve the generalizable utility of this work for other investigators.

**Figure 1:**
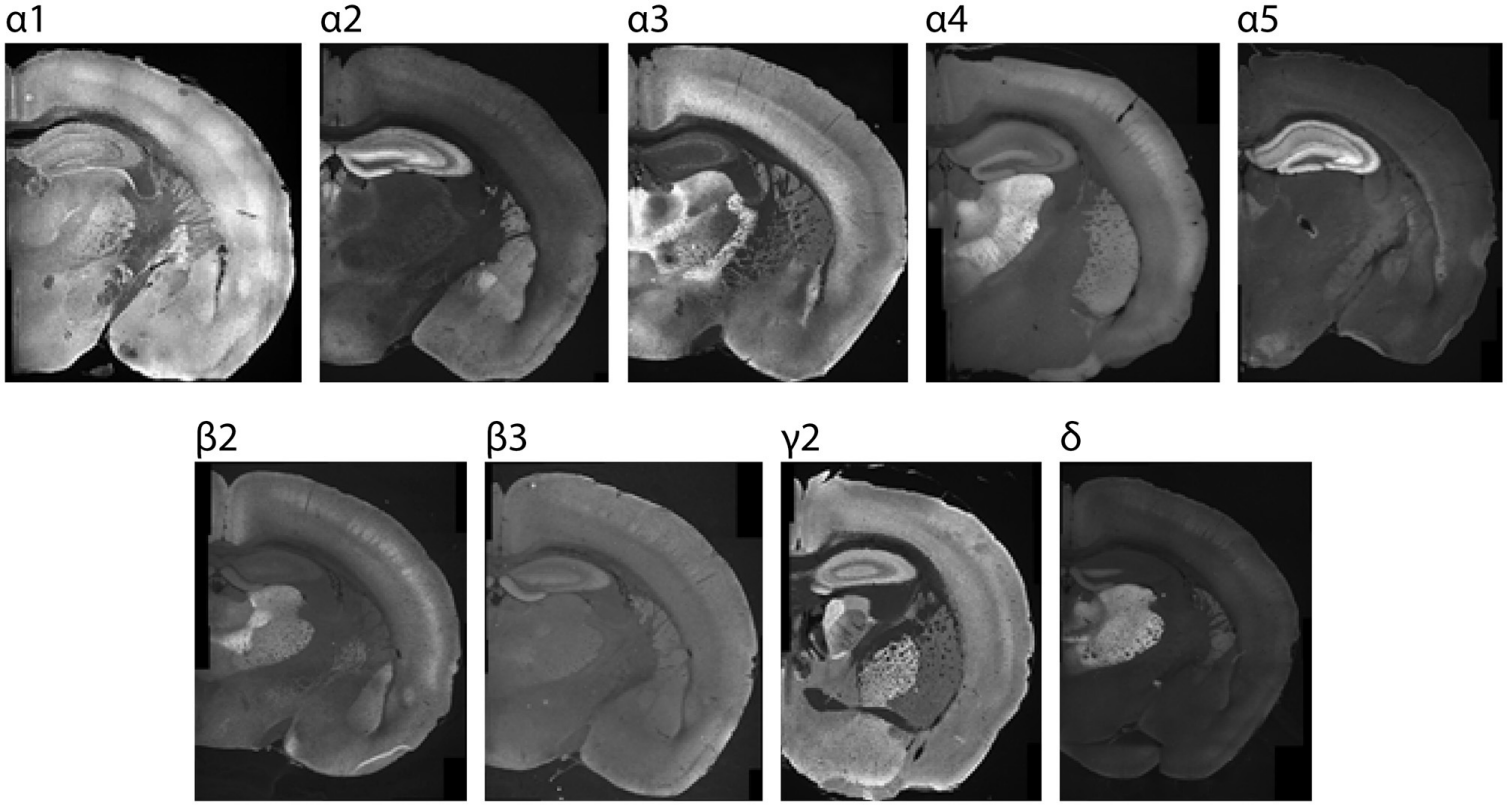
Sample P26 images showing region-specific immunolabeling patterns of GABA_A_R subunits in the CNS. Regions with the highest expression were used for setting acquisition settings and the maximum grading threshold for quantifying subunit expression within cortex.

When validating our antibodies, we first screened for subunit specificity using recombinant receptors expressed in HEK 293T cells (Lagrange et al., 2007). Whole cell protein extracts (data not shown) and plated HEK cells (Supplementary Figure 1) were analyzed by immunoblot to find isoform-specific anti-GABA_A_R antibodies which detect the appropriately sized protein band for the target protein without cross-reactivity with off-target subunit isoforms. We then further confirmed antibody sensitivity and specificity of non-denatured proteins by immunostaining young adult mouse brain tissue, selecting antibodies that labeled regionally specific patterns of subunit-specific expression found in previously published reports (Pirker et al., 2000; Hortnagl et al., 2013) (Figure 1). The α4 immunostains were sometimes associated with a non-specific punctate signal that we could not entirely prevent, which presented as a patchy, inconsistent signal that was equally present in tissue known to lack expression of α4, such as postnatal white matter. While most antibodies have well-defined patterns of high/low expression, this is not true for the more ubiquitously expressed β3, γ2 subunits, so these antibodies were validated using embryonic brain slices from knockout mice that do not express those proteins, using tissue that was generously provided by Jing-Qiong Kang's lab. We also confirmed specificity of α3 and KCC2 expression using knockout mice. The resulting list of validated antibodies used in our study is presented in Supplementary Table 1.

### HEK293T transfection and immunocytochemistry

Human embryonic kidney cells (HEK293T) were maintained in culture and transfected as previously described (Lo et al., 2014). Cells were transfected with a combination of cDNAs, each containing the sequence for one of the rat GABA_A_R mRNA. These were done using Fugene with equimolar amounts (1 μg) of each cDNA. For Western blot (WB), cells were then harvested, and protein extracted as described below. For immunocytochemistry, cells were harvested and then replaced at 10K cells per well in a 24-well plate. They were then cultured overnight and then processed for immunocytochemistry. We tested a number of fixation techniques, including methanol, 4% PFA × 5 min, and 4% PFA + 4% sucrose for 15 min. We also tested multiple blocking conditions, including milk, BSA, or donkey/horse serum, with or without Triton X-100. Based on these results, we chose 4% PFA × 5 min, blocked in 10% donkey serum + 0.3% Triton X-100, then overnight incubation with the primary antibody without Triton X-100 at 1:250 to 1:500. These were then stained with the appropriate secondary antibody, as described in the immunofluorescence section. Wells were then washed three times in PBS, followed by imaging at 10 × with an upright Zeiss microscope.

### Western blots

Tissue was homogenized with a sonicator (QSonica) in a modified RIPA buffer containing (50 mM Tris-HCl pH 7.4, 150 mM NaCl, 1% NP-40, 0.2% sodium deoxycholate, 1 mM EDTA) with 10 μL/mL protease and phosphatase inhibitors (Sigma). Protein concentration was determined with the Bradford assay (Bio-Rad), and samples were diluted to a final concentration of 1 to 3 μg/μL in Laemmli loading buffer (Biorad) containing β-mercaptoethanol. Heat denaturation was skipped due to the delicate nature of transmembrane proteins like GABA_A_R subunits. Samples and protein ladder (Cytiva RPN800E) were loaded onto a 10% SDS gel (Invitrogen) and run at 75–85 V for 2.5–3 h. Proteins were transferred in a Tris-glycine transfer buffer (19.2 mM Glycine, 7.5 mM Tris-base, 20% methanol) onto Immobilon-FL PVDF membranes (Millipore) by applying 100 V for 1.5 h. Membranes were then blocked in TBS with 0.1% Tween-20 (TBS-T) with 4% milk for 1 h at room temperature. Membranes were cut into portions containing E6AP (top) and actin/GABA_A_R protein (bottom) and incubated with respective primary antibodies in 5% BSA in TBS-T overnight at 4°C. Primary antibodies were the same as those used for immunofluorescent staining of brain slices. Membranes were washed at least three times for 10 min in TBS-T and then incubated with fluorescent secondary antibodies in TBS-T for 2 h at room temperature or overnight at 4°C. Membranes were then washed three times for at least 10 min with TBS-T. Imaging was performed on a LI-COR Odyssey fluorescence scanner, protein expression was quantified with Odyssey imaging software, and further analyzed with MS Excel and GraphPad Prism. E6AP and actin were both evaluated as loading controls, after which E6AP was used to normalize all WB expression data for loading differences. The ratios of GABA_A_R/E6AP were averaged, and the mean ± SEM were plotted vs. age. Differences in antibody affinity preclude direct comparisons of protein amounts among different subunits. Therefore, the point of maximal expression for each subunit was used to normal WB data for plotting. Data are depicted as arbitrary units (a.u.) relative to the maximal data point.

To minimize experiment-to-experiment variability, several WBs containing a full range of ages were run, probed for a single GABA_A_R subunit, and analyzed simultaneously. Typically, at least three measurements from three different mouse samples per developmental timepoint per antibody were collected. On a few occasions, we ran additional gels without the complete range of ages. This was due to either needing additional ages to characterize periods of rapid change, or because there was a loading error with a particular protein sample. In these uncommon cases, we included at least 2 protein samples from previously run gels to confirm consistency in our results. The anti-γ2 antibody here recognized both the long and short splice variants of this protein, but their sizes were too similar to discriminate. Examples of GABA_A_R subunit and E6AP WB bands obtained from cortical samples are shown in Supplementary Figure 2. Due to low levels of α4 expression in cortex during development, P36 thalamic and cortical samples were run as controls.

To perform statistical analysis on WB data, expression data was binned into E13.5-P3, P5-P10, and P12-P26 bins to represent generalized developmental stages and avoid type II error from having too many groups. Significance was determined using a one-way ANOVA with Šídák's multiple comparisons test between each of the three bins.

### Immunofluorescence

The slides chosen for cortical staining included coronal sections containing somatosensory cortex. In postnatal tissue, this was further defined as coronal sections containing somatosensory and barrel cortex, and dorsal hippocampus. While not reported here, this plane of coronal sections also allowed us to visualize important germinal areas, such as pallial ventricular and subventricular zones (SVZ), median ganglionic eminence (MGE), intermediate zone (IZ), and postnatal hippocampus, thalamus, and basal ganglia. These postnatal areas were chosen based on strong subunit-specific expression for each. This allowed us to quality control for antibody specificity and qualitatively assess relative expression intensity from run to run. These results were used to conservatively optimize image acquisition parameters before imaging. For example, thalamic α3 expression is high in the reticular nucleus, but absent in ventrobasal thalamus. In contrast, α4 and δ expression are high in the ventrobasal thalamus, but not in the reticular nucleus. Specific cortical areas were identified using Prenatal Mouse Brain Atlas (Schambra and Schambra, 2008) and Chemoarchitectonic Atlas of the Developing Mouse Brain (Jacobowitz and Abbott, 1997) for E13.5 and E15.5 mice, Atlas of the Developing Mouse Brain at E17.5, P0, and P6 (Paxinos, 2007) for perinatal mice, and The Mouse Brain in Stereotaxic Coordinates (Paxinos and Franklin, 2004) for sections from mice P12 and older.

In order to minimize inter-run variability, immunolabeling was performed in batches that included multiple age groups: E13.5, E15.5, E17.5, P1, P5, P12, and/or P25/26. Immunolabeling was done between 4–10 times for each antibody, totaling tissue from 3–8 different mice for each age group. We also included a few slides with no primary antibody as control slides. Slides were labeled and circled around the tissue with the hydrophobic Pap Pen, dried at room temperature for 30 min, then washed (1 × PBS, 0.2% Triton X-100) for 5 min. Slides were then blocked in blocking buffer (1 × PBS, 0.2% Triton X-100, 10% donkey serum) for 1 h, then incubated with the appropriate primary antibody in blocking buffer at 4°C for two nights. Slides were washed 3 times for 5 min, then incubated with the appropriate secondary antibody in blocking buffer for 2 h at room temperature or overnight at 4°C. Sections were washed 3 times for 5 min, mounted and cover slipped with VectaShield HardSet Antifade Mounting Medium with DAPI (Vector Labs, Burlingame, CA), dried for 30 min at room temperature, and stored at 4°C. Images were acquired within 2 to 3 weeks of mounting.

### Microscopy

Stained brain sections were imaged using a Leica DM 6000 epifluorescent microscope equipped with a DFC 365 FX digital camera (Leica, Buffalo Grove, IL). Images were acquired using 5 × and 10 × objectives. Acquisition settings for each antibody were determined using normative regions of interest (ROIs) from P26 brains (Figure 1), and the settings within each run were kept consistent for each subunit. Normative ROIs with maximal subunit expression include: α1: ventrobasal thalamus, α2: dentate gyrus molecular layer, α3: thalamic reticular nucleus, α4 and δ: dentate gyrus molecular layer, α5: CA3 of hippocampus, γ2: globus pallidus, β2: ventrobasal thalamus, and β3: dentate gyrus molecular layer. The grid images were stitched using Fiji Image J stitching plugin (Preibisch et al., 2009): stitching-grid/collection stitching, 30% overlap, maximum intensity fusion method with subpixel accuracy. The fused images were saved as 8-bit TIFF files. Post-stitching modifications included adjustments for contrast and were carried out for display purposes only using Image J. A subset of images was also acquired using higher magnification (20 × or 40 ×), as indicated in the text.

### Image analysis

Brain regions were identified using dedicated atlases of embryonic, perinatal, and adult mouse brain (Jacobowitz and Abbott, 1997; Paxinos and Franklin, 2004; Paxinos, 2007; Schambra and Schambra, 2008). When needed, marginal zone (MZ) and subplate (SP) were further identified (Bayer and Altman, 1990). The most superficial layer of the cortical plate (CP) was defined as MZ before P1, and then as layer 1 postnatally. Cortical layers were defined using DAPI staining of our tissue. We then performed semi-quantitative grading of expression based on age and cortical layers by quantifying the mean intensity in at least three randomly selected regions for each region/layer/age. These numeric results for all ages and cortical layers within each run of IHC were collated to determine the distribution of our results. This information was then used to determine the percentile ranks of each data point and were the basis for initial grades (e.g., >90% percentile was considered a “+++”). Multiple investigators (AHL, AH, KZ, and ZK) then reviewed these grades and the original source images from multiple runs to form a consensus semi-quantitative grading. Each experimental run included E13.5 to P26 tissue, and the ROIs chosen as the normative reference areas of highest expression were assigned a value of 5, while postnatal white matter as 0. Images from at least three animals per timepoint were used in making these assessments. Some subunits had a pattern of expression that was stronger in either the upper or lower portion of a particular layer. This occurred most commonly with L5. In these situations, we report the upper portion as L5a, the lower portion as L5b. This distinction is based purely on the pattern of expression and may not exactly match sublayers reported in the literature that are based on other patterns of expression or physiology. Composite, multi-age figures were typically created from tissue run at the same time to more accurately convey expression-intensity differences over time.

### Laminae in figures and grading tables

In figures, divisions of adult and transient development-specific lamina were defined based on the DAPI signal and were then superimposed on greyscale IHC images. Grading tables used definitions of adult laminae and the transient subplate for ease of tracking laminar changes within a single row. At E13.5-P1, MZ corresponds to L1 in the grading table. CP corresponds to L2-L4 in the grading table for E15.5-P1. Expression for most subunits was ubiquitous in the CP, but when certain banding was observed at the bottom of the CP, this was distinguished as L4-specific signal at these early stages. For some images at E15.5 when no expression differences were seen between CP, L5, and L6, we did not distinguish these laminae and labeled them all as CP. For images at E13.5, CP corresponded to L2–L6 in the grading table.

## Results

We started our investigation by validating GABA_A_R subunit specificity for a panel of antibodies for α1-5, β2-3, δ, and γ2 *in vitro* in HEK cells expressing different GABA_A_R combinations (Supplementary Figure 1), and showing that regional IHC expression at P26 (Figure 1) matched previous reports (Hortnagl et al., 2013). Next, we used the validated antibodies to create a developmental profile of GABA_A_R subunit expression in cortex at different timepoints. Our approach included (1) a quantitative comparison of expression changes associated with each developmental stage by Western blot (WB) across a detailed timeline with statistical analysis performed between three generalized stages (E13.5-P3; P5-P10; P15-P25); and (2) a complimentary layer and region-specific analysis of expression by immunohistochemistry (IHC). Using IHC, we generated a large dataset of immunolabeled tissue, which we used for semi-quantitative grading of laminar expression differences as the development progresses. We designed our semi-quantitative approach (refer to Methods) based on seminal expression studies in our field (Pirker et al., 2000; Hortnagl et al., 2013; Stefanits et al., 2018), which is arguably the most objective approach to quantifying IHC expression while taking into account staining variability and other limitations of IHC. We found that each subunit showed distinct temporal and layer-specific patterns of expression, which are discussed below.

### GABA_A_R α1

**Figure 2:**
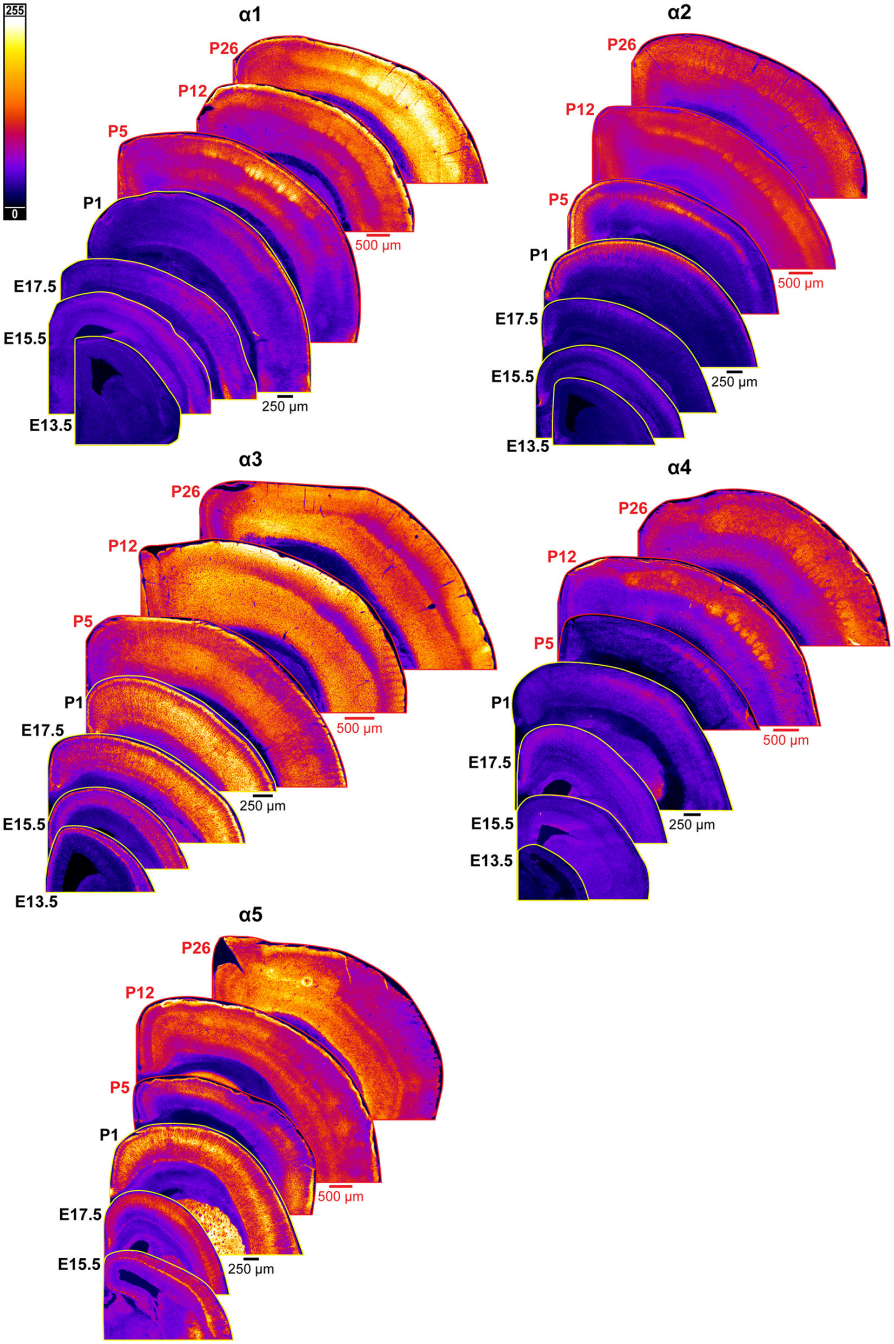
Expression of GABA_A_R α1-5 subunits in developing cortex. Coronal sections are overlaid from embryonic day E13.5/E15.5 on the bottom left to postnatal day 26 on the top right separately for each α subunit. All sections are oriented from ventral bottom to dorsal top, with lateral cortex on the right. Separate spatial scaling has been used for sections E13.5-P1 (black scale bar) and P5-P26 (red scale bar), separately for each subunit. Signal is represented using a subunit-specific heat map lookup table to highlight differences in regional expression. Heatmap intensity scaling is shown by the bar in top left.

**Figure 3:**
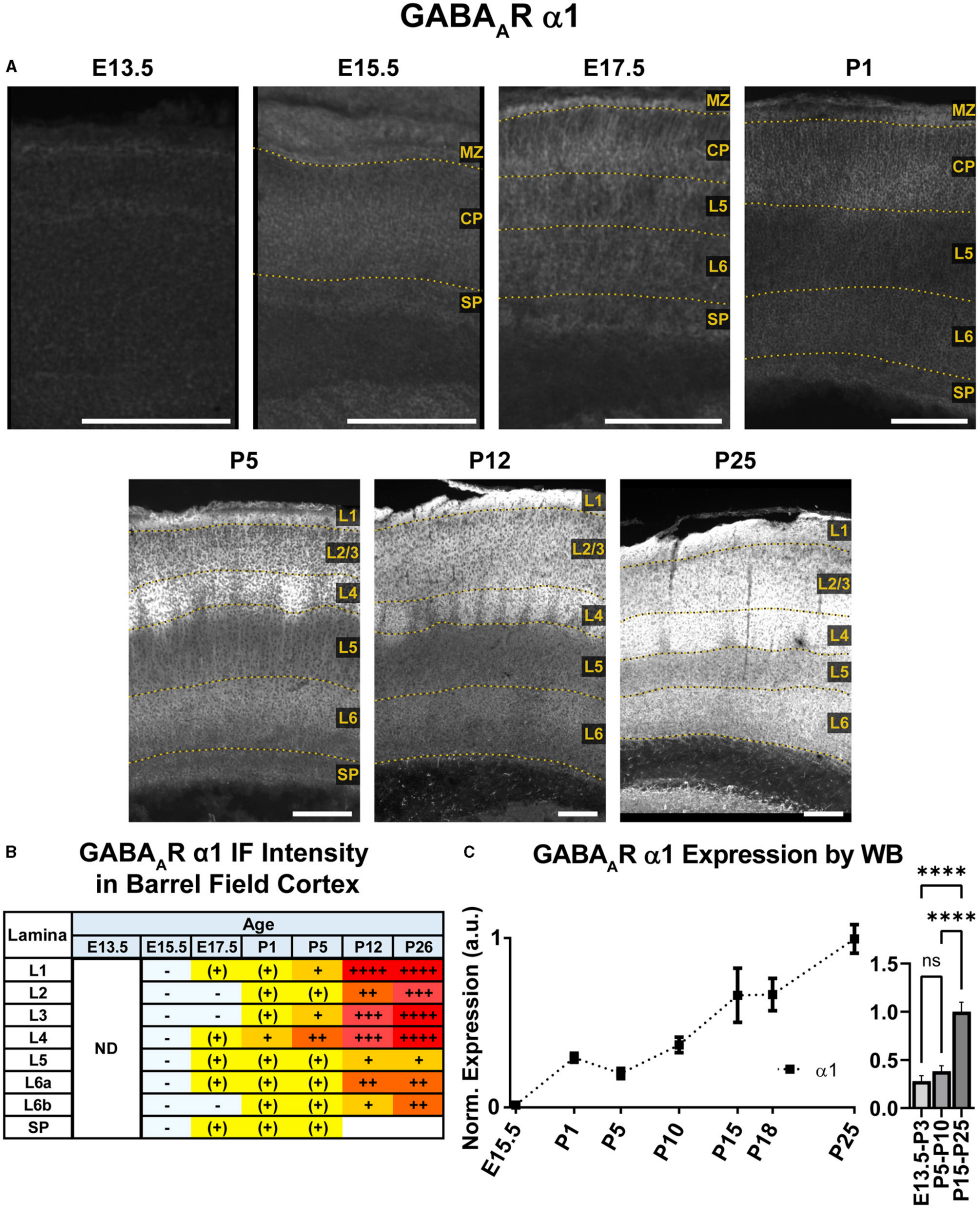
GABA_A_R α1 expression in barrel field cortex from embryonic age to maturity. **(A)** Exemplar images of α1 immunoreactivity in barrel field cortex across development. Scale bar = 250 μm. **(B)** Grading of immunofluorescence intensity across cortical lamina, where + + ++ is the maximal α1 signal that could be detected in brain at P26. **(C)** Quantification of GABA_A_R α1 protein in cortical samples by Western blot (WB), with a timeline of all points on the left and binned stages tested for significance on the right. ^****^P < 0.0001 by one-way ANOVA with Šídák's multiple comparisons; n in mice/age: 3/E15.5, 8/P1, 3/P5, 4/P10, 4/P15, 4/P18, and 4/P25. L1–L6, layer 1–layer 6; MZ, marginal zone; SP, subplate; CP, cortical plate; ND, not determined; also refer to “Lamina in Grading Table and Figures” section in Methods.

An overview of α1-5 subunit expression is shown in Figure 2. Cortical immunoreactivity of α1 on WB was generally low in embryonic and early postnatal period, but steadily rose to prominent and then high levels in the late postnatal period that showed a statistically significant difference from earlier expression (Figure 3, ^****^P < 0.0001 P15-P25 vs. earlier ages). Our IHC experiments corroborated this trend and revealed significant regional and lamina-specific differences (Figures 2, 3). At E13.5, α1 protein expression was essentially absent from the developing cortex and underlying regions, and only very low α1 levels were seen at E15.5. Somewhat higher expression in the cortex and subplate was evident at E17.5 and P1, primarily in marginal zone (MZ)/layer 1 (L1) and layer 4 (L4). At these ages, future somatosensory cortex could be distinguished from adjacent regions by elevated α1 expression in L4. While α1 was low in other layers at E17.5-P1, there were clear α1-positive putative dendrites in L2 that appeared to arise from L4 and end in dendritic tufts in L1 (Figure 2) in a fashion similar to previously reported (Paysan et al., 1997; Paysan and Fritschy, 1998).

The overall α1 expression levels rose dramatically in most cortical areas at P5 but retained a layer-specific pattern. Increased α1 expression in L2/3 made it impossible to distinguish the aforementioned L2/3 dendrites by P5. However, the highest expression remained in L1 and L4, as well as somewhat increased expression at the boundary of L5b/L6a. This gave the appearance of alternating bands of high expression in L1, L4, and L5b/L6a boundary with lower expression in-between. Expression at P12 and P26 continued to rise throughout cortex, becoming most prominent in L1-4 and highest in L3/4 of barrel cortex. Expression within L5/6 was comparatively lower at these ages, but consistently higher in L6 than L5.

### GABA_A_R α2

**Figure 4:**
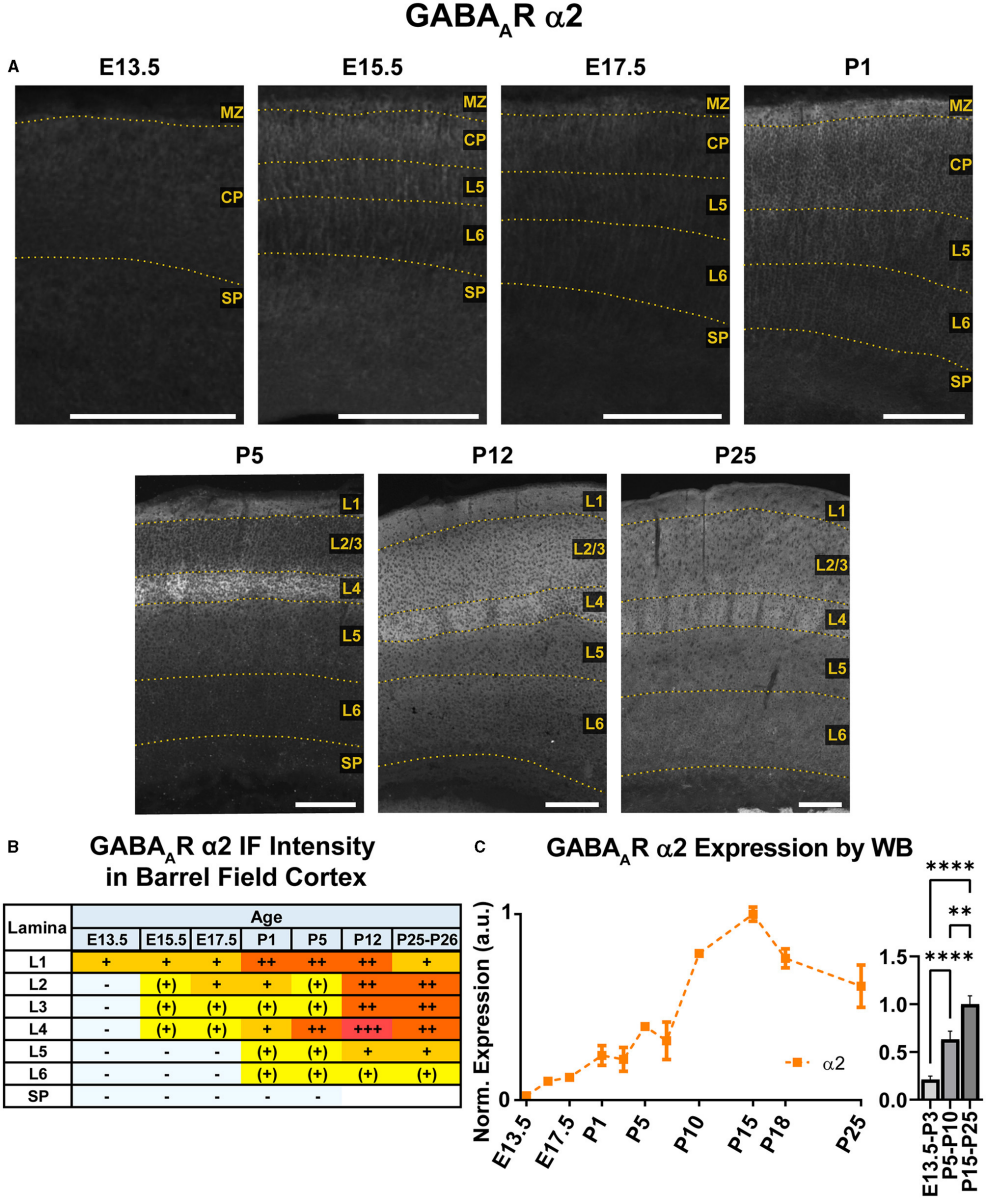
GABA_A_R α2 expression in barrel field cortex from embryonic age to maturity. **(A)** Exemplar images of α2 immunoreactivity in barrel field cortex across development. Scale bar = 250 μm. **(B)** Grading of immunofluorescence intensity across cortical lamina, where + + ++ is the maximal α2 signal that could be detected in brain at P26. **(C)** Quantification of GABA_A_R α2 protein in cortical samples by Western blot, with a timeline of all points on the left and binned stages tested for significance on the right. ^****^P < 0.0001, ^**^P < 0.01 by one-way ANOVA with Šídák's multiple comparisons; n in mice/age: 3/E13.5; 3/E15.5, 3/E17.5, 6/P1, 4/P3, 5/P5, 5/P7, 4/P10, 3/P15, 4/P18, and 5/P25. L1–L6, layer 1–layer 6; MZ, marginal zone; SP, subplate; CP, cortical plate; also refer to “Lamina in Grading Table and Figures” section in Methods.

**Figure 5:**
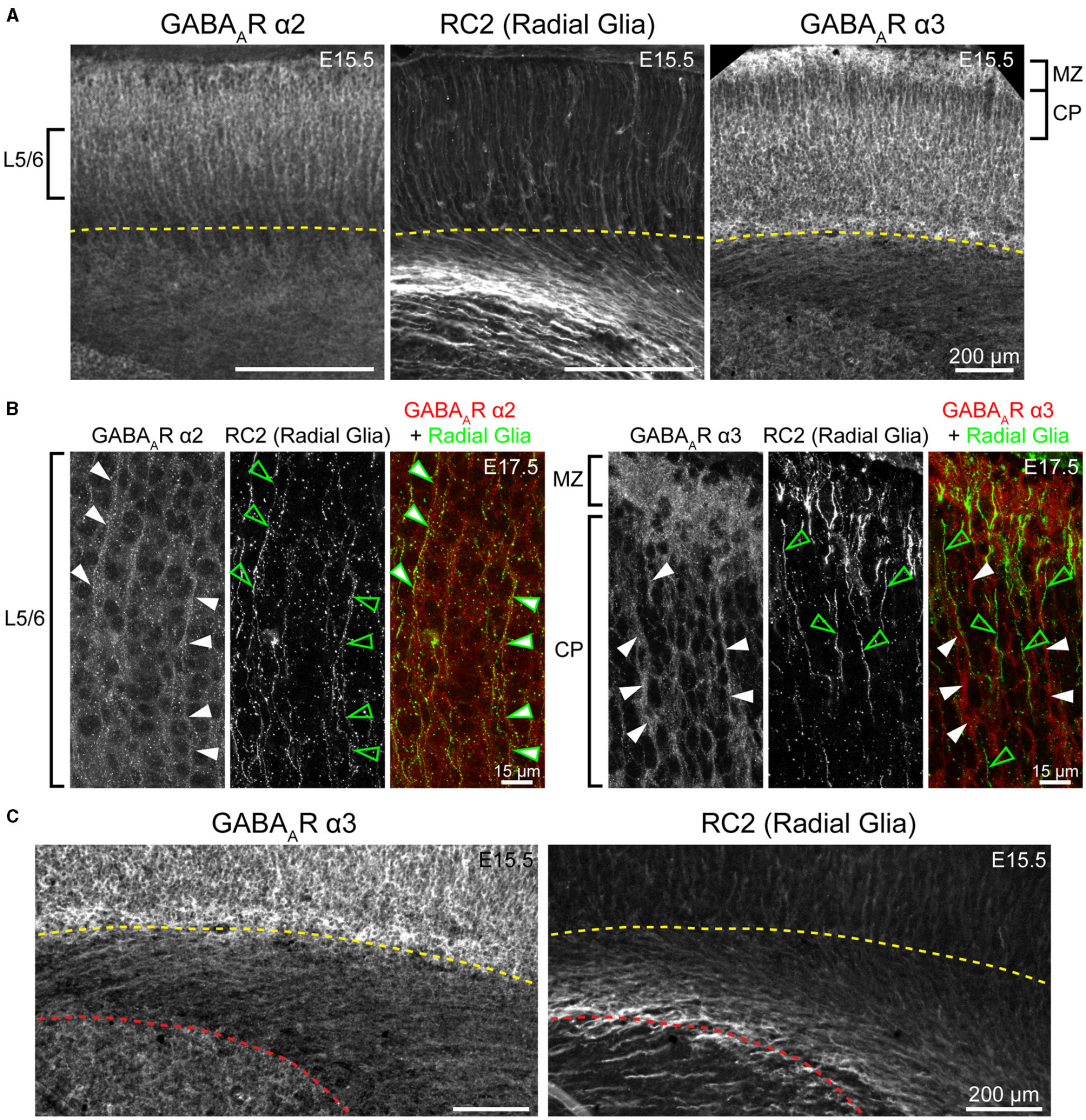
Fibers of radial glia co-localize with GABA_A_R α2, but not GABA_A_R α3. **(A)** Both GABA_A_R α2 and α3 immunolabeling showed a pattern of ascending cortical fibers, which are prominent in the CP/developing layers 2/3 for α3 (right) and are found throughout the cortical column for α2 (left) in embryonic and early postnatal period. These fibers resemble radial glia marked by RC2 (middle). **(B)** The radial glia marker RC2 co-localized with α2-immunopositive fibers (left, arrows), but not with α3-immunopositive fibers (right, arrows). Note that white arrows denote fibers immunopositive for α2 and α3, while green empty arrows indicate radial glia fibers. **(C)** Subcortical expression of α3-positive fibers has a different pattern than radial glia. The yellow dotted line in **(A, C)** indicates the basal margin of the subplate, while red dotted line in **(C)** indicates the margin between intermediate zone/white matter and the striatum. All images are oriented basal bottom to apical top.

WB analysis showed low α2 expression during embryonic and perinatal periods that greatly increased and peaked at P10-P18, coming down to a moderate level of expression at P25 (Figure 4). Our statistical evaluation showed this as a significant steady increase across development (^****^P < 0.0001 E13.5-P3 vs. P5-P10, ^**^P < 0.01 P5-P10 vs. P15-P25). Our IHC experiments reflected this general trend but detected multiple instances of localized expression throughout development. At E13.5, there was very faint α2 expression in the MZ and subcortical tissue, but not the somatosensory cortical plate (CP) itself. Expression of α2 within the CP began at E15.5, with diffuse α2 expression mostly in the upper layers of CP (L1-3). Interestingly, most of the α2 signal in the lower CP appeared to be a continuation of radial fibers originating in subcortical tissue. This was more prominent at E17.5, with α2-positive radial fibers arising from the intermediate zone, extending through the subplate and then outward toward the cortical surface. These processes are clearly visible in L5/6 but are lost in the generalized α2 expression in more superficial layers. The identity of these fibers is not entirely clear, but they overlap with projections of RC2-expressing radial glia (Figure 5). At P1, the subcortical α2 signal disappears, but these processes are still visible in L5/6 until P5. Within the superficial layers, expression of α2 increased at P1 along a lateral-to-medial gradient, with a narrow strip of expression primarily in L1 in the far lateral somatosensory cortex that widens to include L1-3 in more medial somatosensory cortex and L1-5 in motor cortex (Figure 2).

By P5, there was an abrupt increase in α2 expression in L4, producing a transient pattern of highly restricted α2 expression to L1 and L4. While other GABA_A_R subunit proteins show a specific pattern in L4 somatosensory cortex at this age, the increased α2 expression is more widespread, involving L4 in most cortical areas, such as somatosensory, V1, parietal, auditory, and insular cortices (Figure 2). The one exception remains in adjacent motor cortex, where L4 is poorly distinguished from α2 expression in L2/3. By P12, the α2 expression becomes homogenous in L1-4, though it is still slightly higher in L4. Within L5/6, expression is overall lower, with a subtle band of elevated expression in L5b. This pattern persists at P26, albeit with modestly reduced overall expression. While our WB measurements and previous work (Fritschy et al., 1994) indicate a peak expression during the second or third postnatal week, we did not see a strong drop in tissue immunoreactivity within the somatosensory cortex at P26.

### GABA_A_R α3

**Figure 6:**
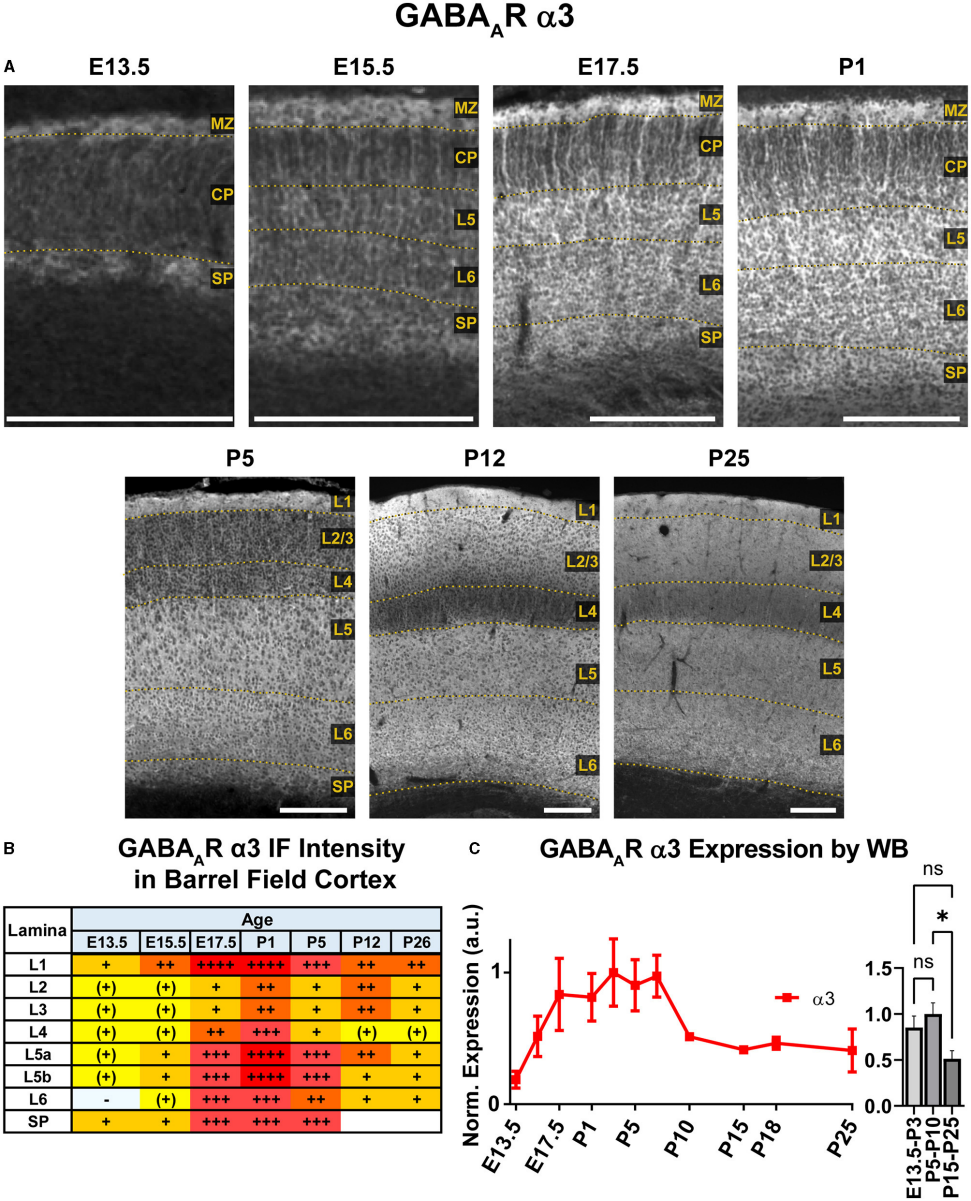
GABA_A_R α3 expression in barrel field cortex from embryonic age to maturity. **(A)** Exemplar images of α3 immunoreactivity in barrel field cortex across development. Scale bar = 250 μm. **(B)** Grading of immunofluorescence intensity across cortical lamina, where + + ++ is the maximal α3 signal that could be detected in brain at P26. **(C)** Quantification of GABA_A_R α3 protein in cortical samples by Western blot, with a timeline of all points on the left and binned stages tested for significance on the right. *P < 0.05 by one-way ANOVA with Šídák's multiple comparisons; n in mice/age: 3/E13.5; 3/E15.5, 3/E17.5, 6/P1, 4/P3, 6/P5, 6/P7, 4/P10, 3/P15, 4/P18, 6/P25. L1-L6, layer 1-layer 6; MZ, marginal zone; SP, subplate; CP, cortical plate; also refer to “Lamina in Grading Table and Figures” section in Methods.

WB analysis showed high cortical levels of α3 during embryonic development, steadily increasing from E13.5 with a plateau between E17.5-P7, then decreasing to a lower plateau at P10 onwards that was significantly different from earlier postnatal expression (Figure 6; ^*^P < 0.05 for P15-P25 vs. P5-P10). Our IHC analysis showed a highly lamina-specific pattern of α3 expression at all timepoints (Figures 2, 6). Expression of α3 first appeared in the MZ and subplate at E13.5 with lower levels throughout the CP. The subcortical band of α3 actually included the subplate and adjacent cell-poor zone below the CP, and this was present throughout the embryonic ages. Over development, this subplate-specific α3 expression merged with expression in L6 and became indistinguishable from L6 by P5. At E15.5, expression of α3 increased in lower portions of the CP (L5/6) with what appeared to be cytoplasmic expression of many cells superimposed on a more diffuse pattern (details best seen in Figure 5A). In contrast, there was little intrinsic α3 expression in the upper CP, although there were clear radial fibers extending from L5/6 that appeared to end in intensely stained dendritic tufts in L1 (Fritschy et al., 1998). This pattern of robust α3 in the lower CP with presumed dendrites in L2-4 persisted until P5. Compared to α2-positive fibers, these α3-stained fibers differed in a number of features. While α2-positive fibers were narrow and appeared primarily in deep cortical layers and the intermediate zone, the α3 fibers were found in superficial layers, were much thicker and numerous, and did not co-localize with RC2+ processes of radial glia (Figure 5). At P1 and P5, α3 expression levels continued to rise in cortical layers, making individual layers less distinct. Accordingly, the presumed dendrites were no longer visible in L2/3 after P5, although sparsely distributed α3+ fibers traversing across L4 can be seen as late at P26. Expression of α3 remained highest in the lower levels of cortex (L5/6) with a pattern of strong somatic expression in L5/6 that was superimposed on a lower diffuse level of α3 expression. By P12, this somatic pattern evolved to a more diffuse pattern.

**Figure 7:**
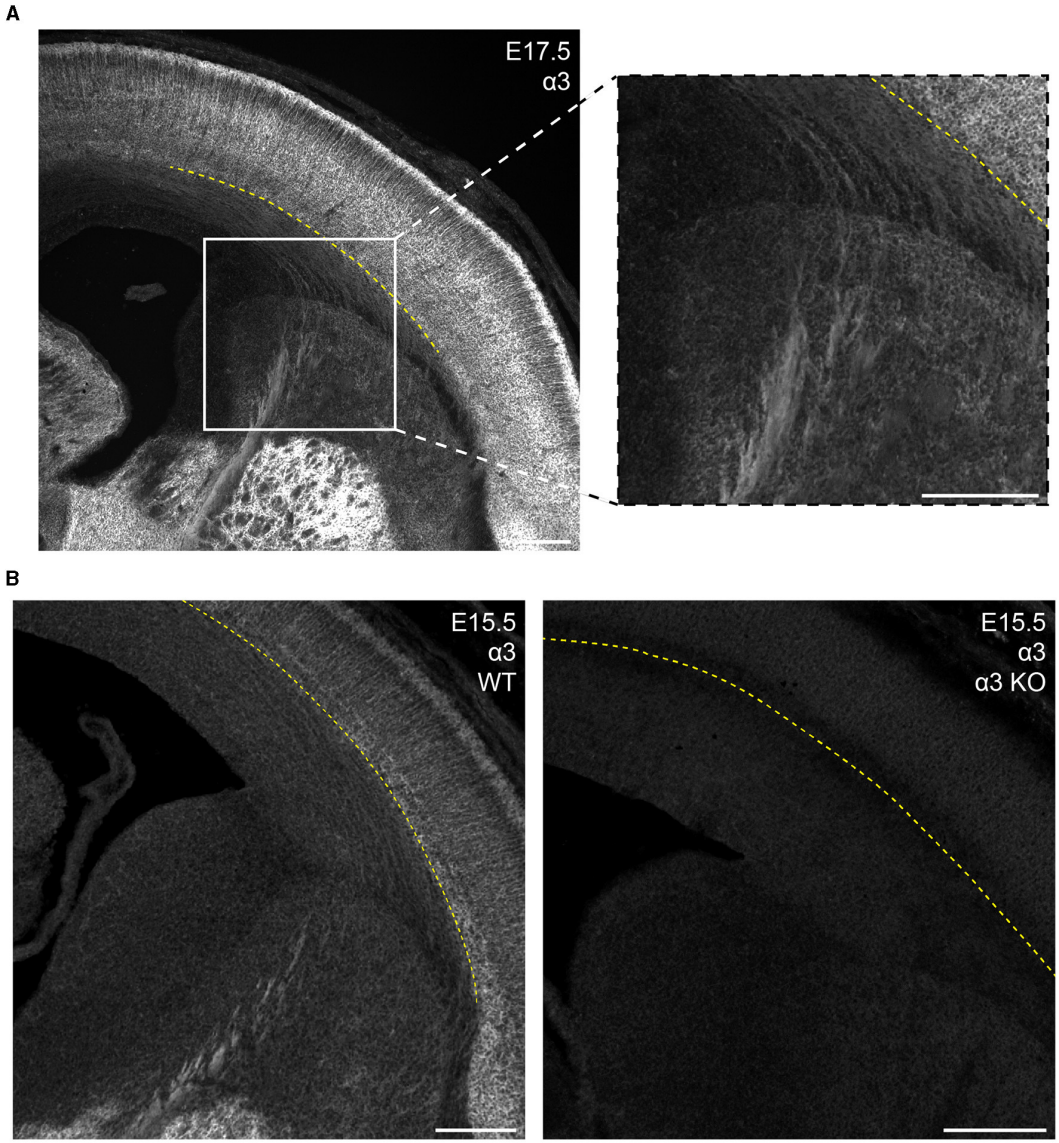
GABA_A_R α3 expression in fibers of the internal capsule during embryonic development. **(A)** E17.5 coronal section showing prominent α3 immunolabeling of internal capsule fibers in the white matter/intermediate zone located below the subplate and running medioventrally through the striatum. These regions of interest are enlarged in the inset. **(B)** α3-immunolabeled fibers are absent in the α3-knockout mouse (α3 KO, right), but consistently present in sections from wildtype mice (left). The yellow dotted line in **(A, B)** indicates the basal margin of the subplate. All images are oriented ventral bottom to dorsal top, with lateral cortex on the right.

In the mature brain, the subcortical white matter is generally devoid of any GABA_A_R subunit protein expression. However, between E15.5 and P1, we saw considerable subcortical α3 expression with apparent fiber tracks running parallel to lower margin of the CP. This pattern was most prominent at E17.5 and not seen in tissue from *Gabra3* knockout mice (Figure 7). This was especially conspicuous in the internal capsule fibers seen running between the thalamus and basal ganglia, as well α3+ fascicles running through the caudate. The subcortical α3 signal was most prominent in the thalamocortical tracks passing through the basal ganglia and into the ventrolateral IZ. This signal was also seen in the external capsule, but not in other tracks like corpus callosum or the anterior commissure.

### GABA_A_R α4

**Figure 8:**
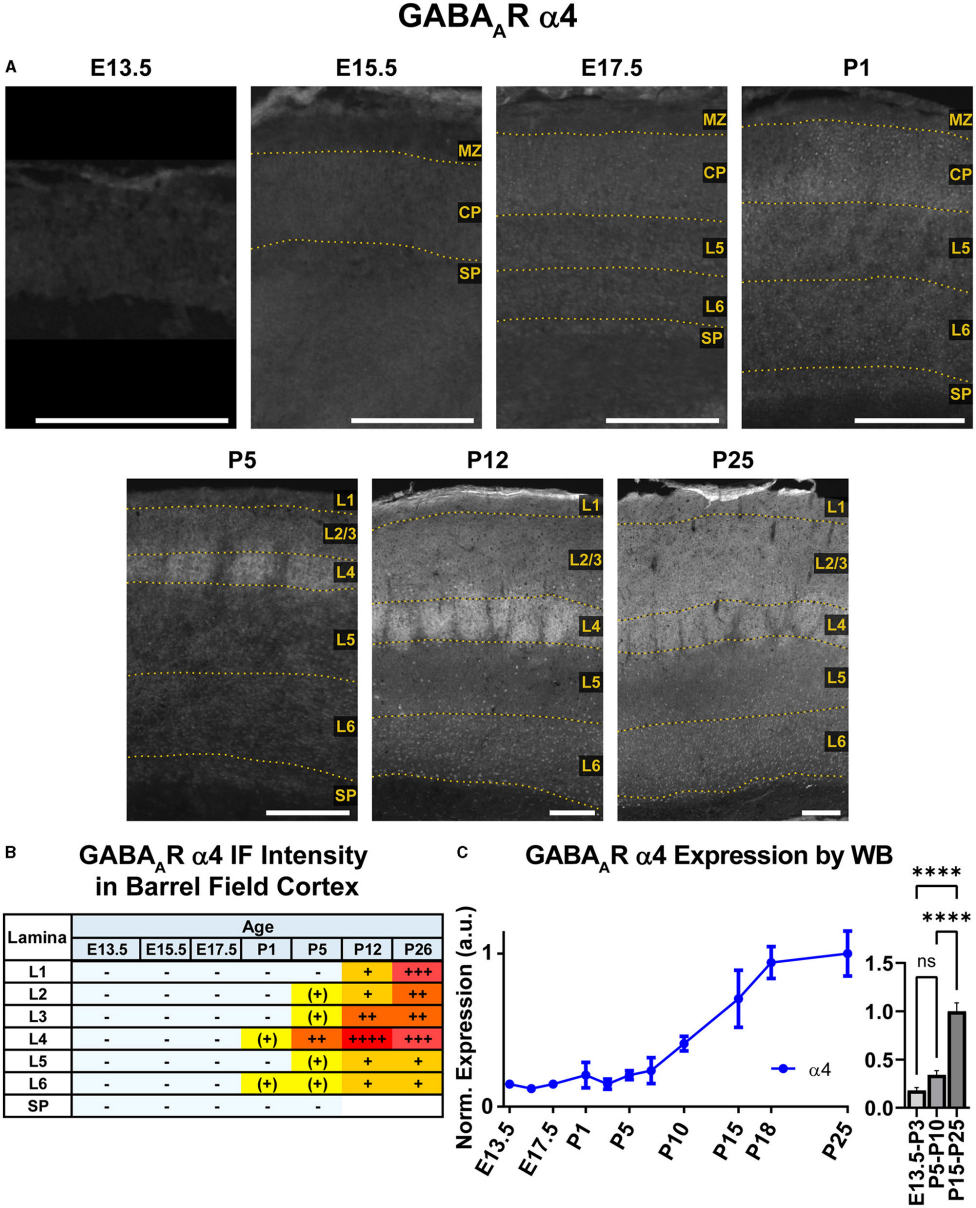
GABA_A_R α4 expression in barrel field cortex from embryonic age to maturity. **(A)** Exemplar images of α4 immunoreactivity in barrel field cortex across development. Scale bar = 250 μm. **(B)** Grading of immunofluorescence intensity across cortical lamina, where + + ++ is the maximal α4 signal that could be detected in brain at P26. **(C)** Quantification of GABA_A_R α4 protein in cortical samples by Western blot, with a timeline of all points on the left and binned stages tested for significance on the right. ^****^P < 0.0001 by one-way ANOVA with Šídák's multiple comparisons; n in mice/age: 3/E13.5; 3/E15.5, 3/E17.5, 6/P1, 3/P3, 4/P5, 3/P7, 6/P10, 3/P15, 5/P18, and 4/P25. L1–L6, layer 1–layer 6; MZ, marginal zone; SP, subplate; CP, cortical plate; also refer to “Lamina in Grading Table and Figures” section in Methods.

Both WB and IHC data showed low cortical α4 expression in prenatal tissue that began to increase at P5, particularly in L1-4 by immunofluorescence (Figures 2, 8), though a significant difference by WB expression was only detected at P15-P25 compared to earlier ages (Figure 8, ^****^P < 0.0001). While α4 mRNA has been reported in embryonic ventricular and sub-ventricular zones (Laurie et al., 1992; Ma and Barker, 1995, 1998), we did not find significant/consistent expression of α4 protein in this area but cannot rule out expression below our level of detection. Similarly, there were very low or negligible α4 levels in the CP and subplate until after P1. By P5, there was distinct α4 expression in L4 of somatosensory cortex, which was slightly more intense in barrels. At P12 and P26, there was increasing α4 expression throughout L1-4 that remained most prominent in L4 of motor and somatosensory cortex. This was especially prominent in the barrels, where α4 expression peaked at P12 and was on par with P26 thalamus, our reference ROI for maximal α4 intensity signal (Figure 1). In L5/6, α4 expression increased slightly at P12 and P26, but remained at a generally low level.

### GABA_A_R α5

**Figure 9:**
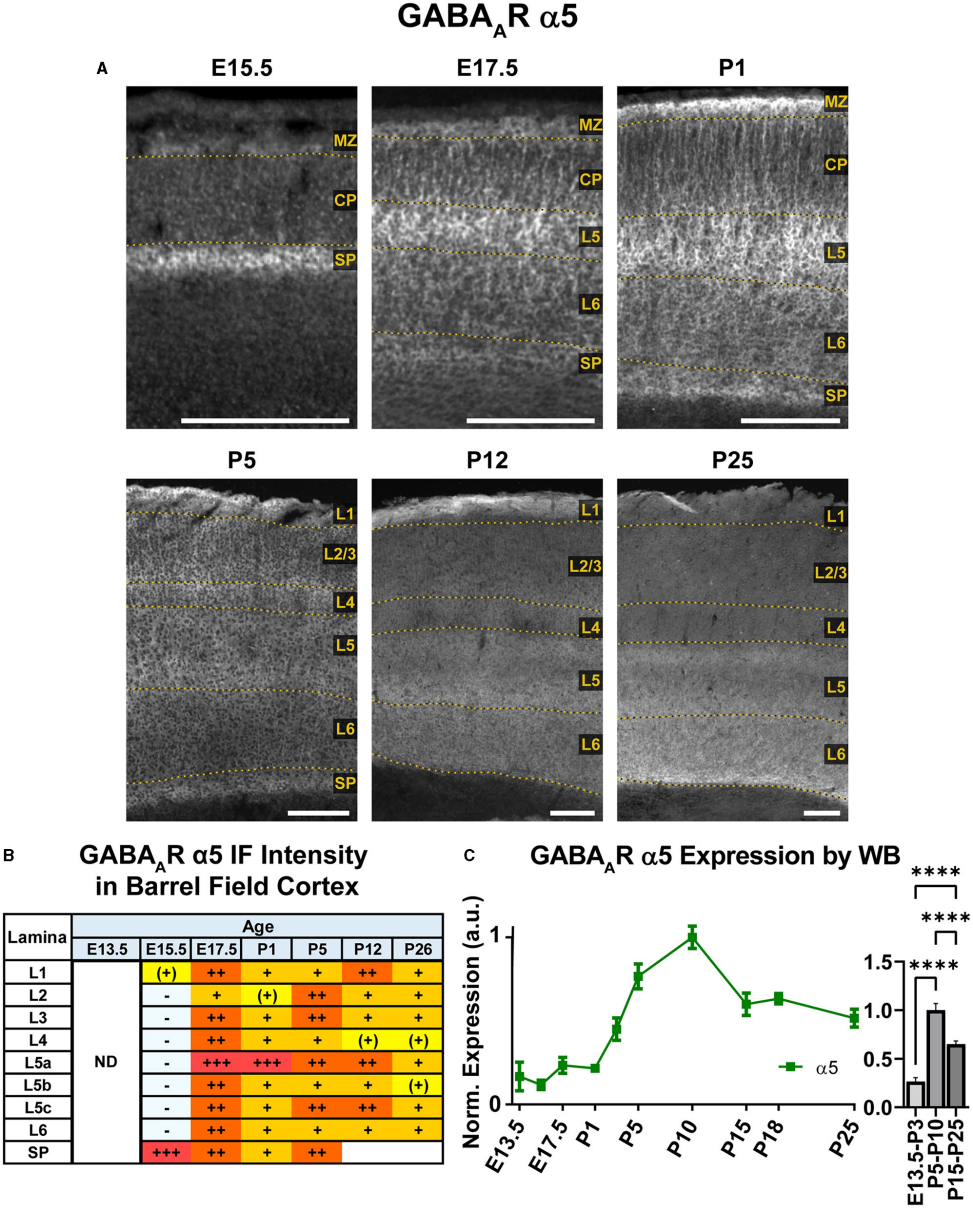
GABA_A_R α5 expression in barrel field cortex from embryonic age to maturity. **(A)** Exemplar images of α5 immunoreactivity in barrel field cortex across development. Scale bar = 250 μm. **(B)** Grading of immunofluorescence intensity across cortical lamina, where + + ++ is the maximal α5 signal that could be detected in brain at P26. **(C)** Quantification of GABA_A_R α5 protein in cortical samples by Western blot, with a timeline of all points on the left and binned stages tested for significance on the right. ^****^P < 0.0001 by one-way ANOVA with Šídák's multiple comparisons; n in mice/age: 3/E13.5; 3/E15.5, 3/E17.5, 3/P1, 3/P3, 4/P5, 3/P7, 5/P10, 3/P15, 7/P18, and 6/P25. L1–L6, layer 1–layer 6; MZ, marginal zone; SP, subplate; CP, cortical plate; ND, not determined; also refer to “Lamina in Grading Table and Figures” section in Methods.

Our WB experiments showed low embryonic and perinatal expression of α5 that suddenly increased and peaked between P1-P10, then decreased to a moderate level from P15 onwards and showed a statistically significant difference between all three developmental stages (Figure 9; ^****^P < 0.0001 E13.5-P3 vs. P5-P10 and P15-P25, ^****^P < 0.0001 P5-P10 vs. P15-P25). In immunostained sections (Figures 2, 9), the first clear expression of α5 was seen at E13.5 in the subplate, but nowhere else. There was a clear lateral to medial gradient, with highest expression in the lateral subplate and claustrum (Figure 2). Prominent α5 in subplate was a persistent feature at all ages studied. There was even a band of higher α5 signal at the lower edge of L6 as late as P26, although we were unable to distinguish L6b vs. subplate after P5 (Viswanathan et al., 2016). At E15.5, α5 was expressed in the MZ, with lower levels in the mid-CP/L5, creating a trilaminar pattern of MZ/L5/subplate. There was relatively little α5 in other layers. At E17.5/P1, α5 expression increased, especially in the upper portions of L5 (L5a). The pattern of expression was mostly as a perisomatic rim around individual cells overlying a more diffuse pattern of α5 expression. There was relatively little α5 in L2-4, although α5+ dendrites from L5 could be seen traversing L2-4 until about P5.

There were significant changes in α5 expression around P5, with most cortical areas showing increased expression in the upper half of cortex, especially L4. However, a distinctly different pattern was seen in the barrel cortex, where α5 virtually disappeared from L2-4. This created an abrupt margin between barrel cortex and the adjacent somatosensory and motor cortices (Figure 2). A similar loss of α5 in L2-4 was seen in primary visual cortex (V1), but not in adjacent cortices (not shown). This pattern in primary sensory cortex and V1 was also reported by Paysan et al. (1997) at P7, who found it was dependent on early sensory input, and could be prevented by ablation of the ventrobasal or lateral geniculate nuclei of thalamus. At around P5, we could also begin to appreciate more complex sublamina in lower cortex, with moderate levels in L5a and L5c/L6a, but lower levels in L5b and L6. This distinct pattern was first seen at P5, but most clearly at P12. The previously noted pattern of somatic α5 superimposed on a diffuse background of α5 persisted in lower cortex until at least P12, with somewhat more numerous cell bodies in L5b and subplate. However, the distinction between somata and neuropil was never as clear as that seen at E17.5/P1. At P26, α5 expression became more diffuse, and individual dendrites and cell bodies became poorly distinguishable. Expression approached moderate levels in all cortical regions and layers, but previous patterns of α5 expression were still apparent; namely, barrel field cortex had lower expression than other cortical regions, deeper cortical layers showed a complex sublaminar expression pattern, and expression in L2/3 was lower than in other layers.

### GABA_A_R δ

**Figure 10:**
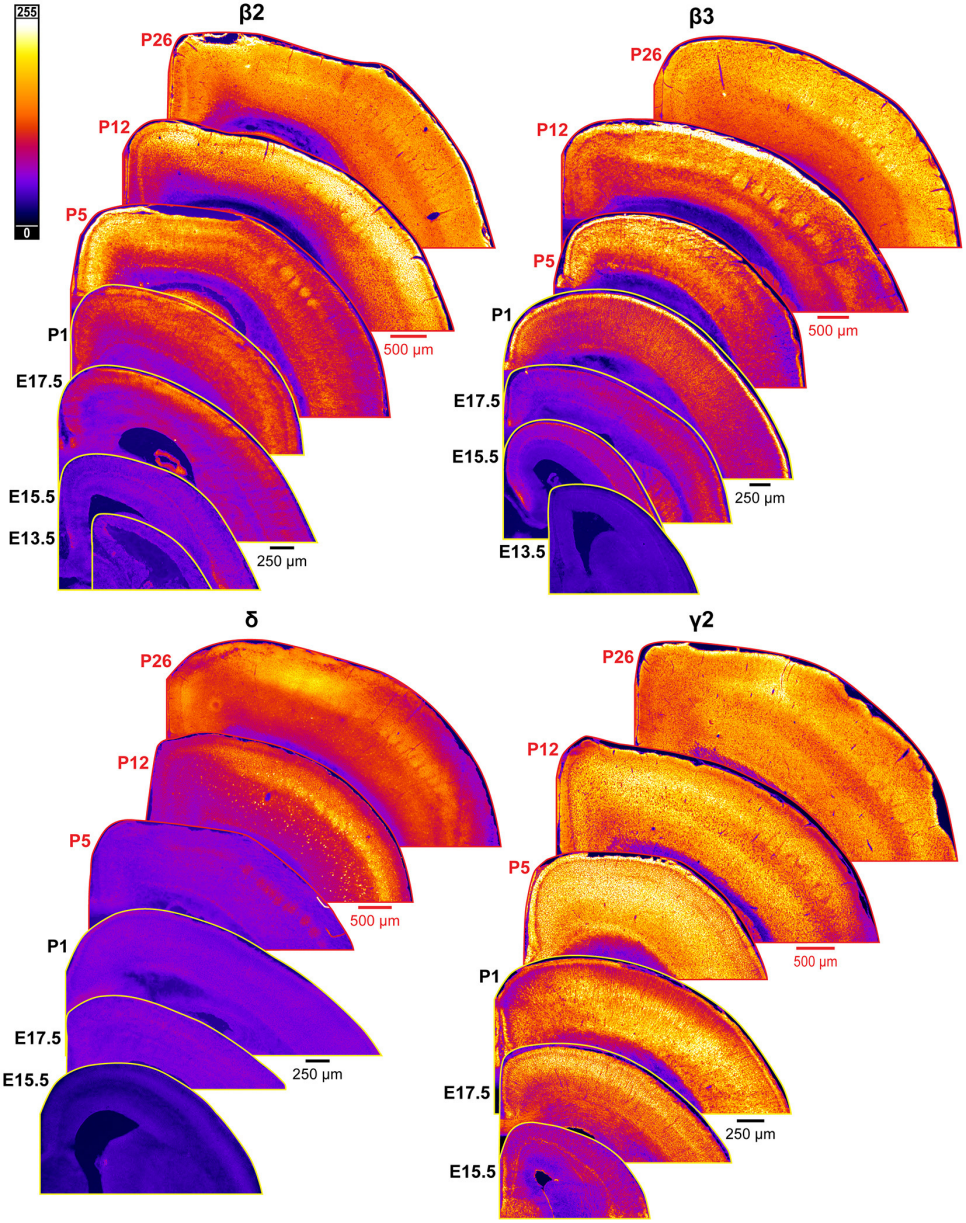
Expression of GABA_A_R β2, β3, δ, and γ2 subunits in developing cortex. Coronal sections are overlaid from embryonic day E13.5/E15.5 on the bottom left to postnatal day 26 on the top right separately for each subunit. All sections are oriented from ventral bottom to dorsal top, with lateral cortex on the right. Separate scaling has been used for sections E13.5-P1 (black scale bar) and P5-P26 (red scale bar), separately for each subunit. Signal is represented using a heat map lookup table to highlight differences in regional expression. Heatmap intensity scaling is shown by the bar in top left.

**Figure 11:**
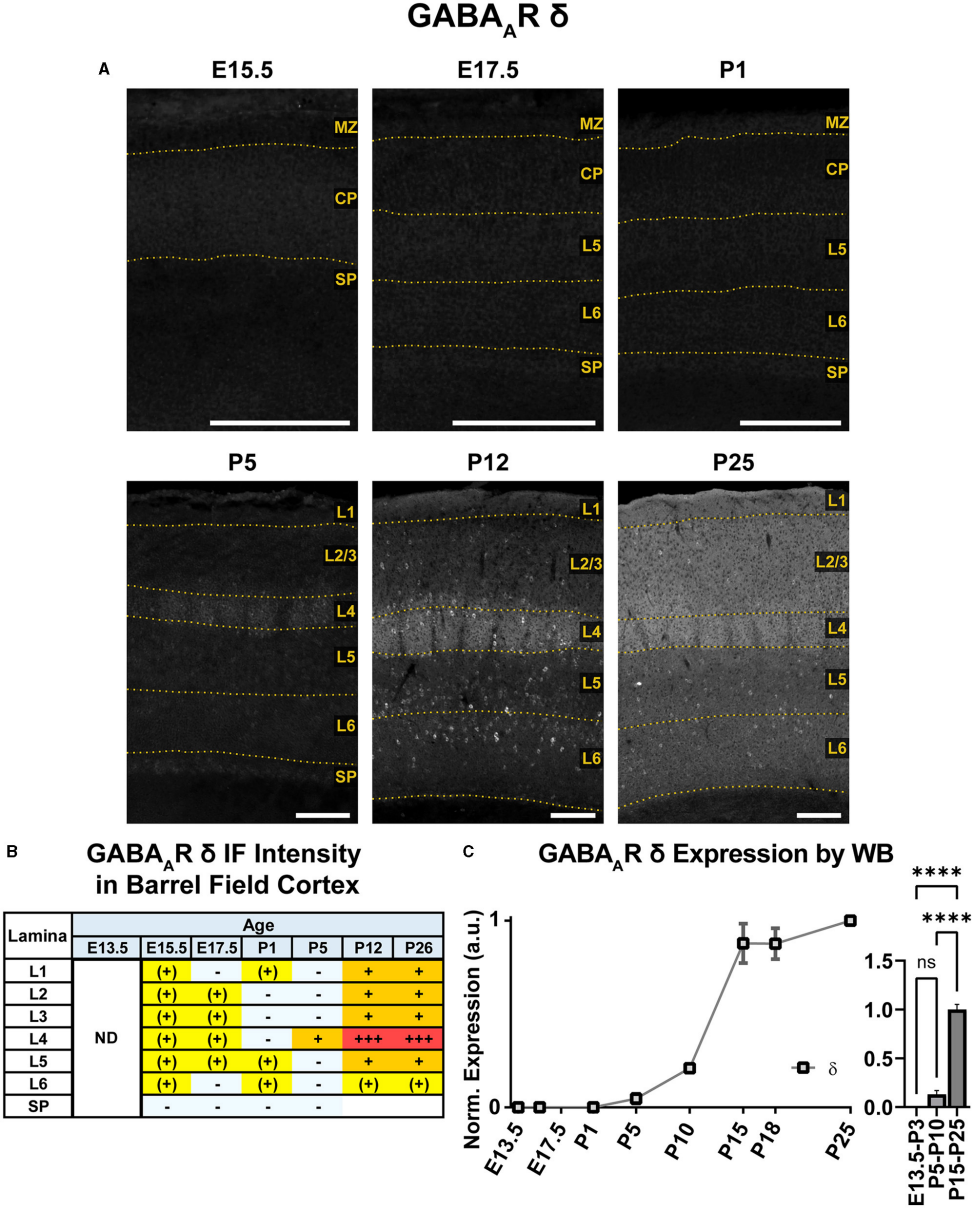
GABA_A_R δ expression in barrel field cortex from embryonic age to maturity. **(A)** Exemplar images of δ immunoreactivity in barrel field cortex across development. Scale bar = 250 μm. **(B)** Grading of immunofluorescence intensity across cortical lamina, where + + ++ is the maximal δ signal that could be detected in brain at P26. **(C)** Quantification of GABA_A_R δ protein in cortical samples by Western blot, with a timeline of all points on the left and binned stages tested for significance on the right. ^****^P < 0.0001 by one-way ANOVA with Šídák's multiple comparisons; n in mice/age: 4/E13.5; 3/E15.5, 4/P1, 4/P5, 3/P10, 4/P15, 5/P18, and 3/P25. L1–L6, layer 1–layer 6; MZ, marginal zone; SP, subplate; CP, cortical plate; ND, not determined; also refer to “Lamina in Grading Table and Figures” section in Methods.

An overview of δ subunit expression is shown together with β2, β3, and γ2 in Figure 10. We detected essentially no embryonic or perinatal expression of the δ subunit in cortex by IHC or WB. By WB, we began to see low cortical expression of δ around P10 that quickly rose to a plateau level at P15-P25, showing a statistically significant difference from earlier ages (Figure 11; ^****^P < 0.0001 P15-P25 vs. earlier ages). However, δ IHC expression in sections (Figures 10, 11) first appeared at P5 as a diffuse signal in L4, most prominently in the barrel cortex, with lower levels in the rest of somatosensory cortex. By P12 and P26, this diffuse pattern of expression increased in all layers, but remained highest in L4 in barrels and relatively low in L5/6.

Superimposed upon this diffuse layer-specific pattern of expression, there were scattered δ+ cell bodies in cortex and hippocampus as early as P5, which became much more evident at P12 and P26. However, these cells were somewhat less visually distinct at P26, likely due to increasing background δ subunit expression. Density of these cells was greatest in L4, L5, and subplate but and very sparse in L1 and lower portions of L6.

### GABA_A_R γ2

**Figure 12:**
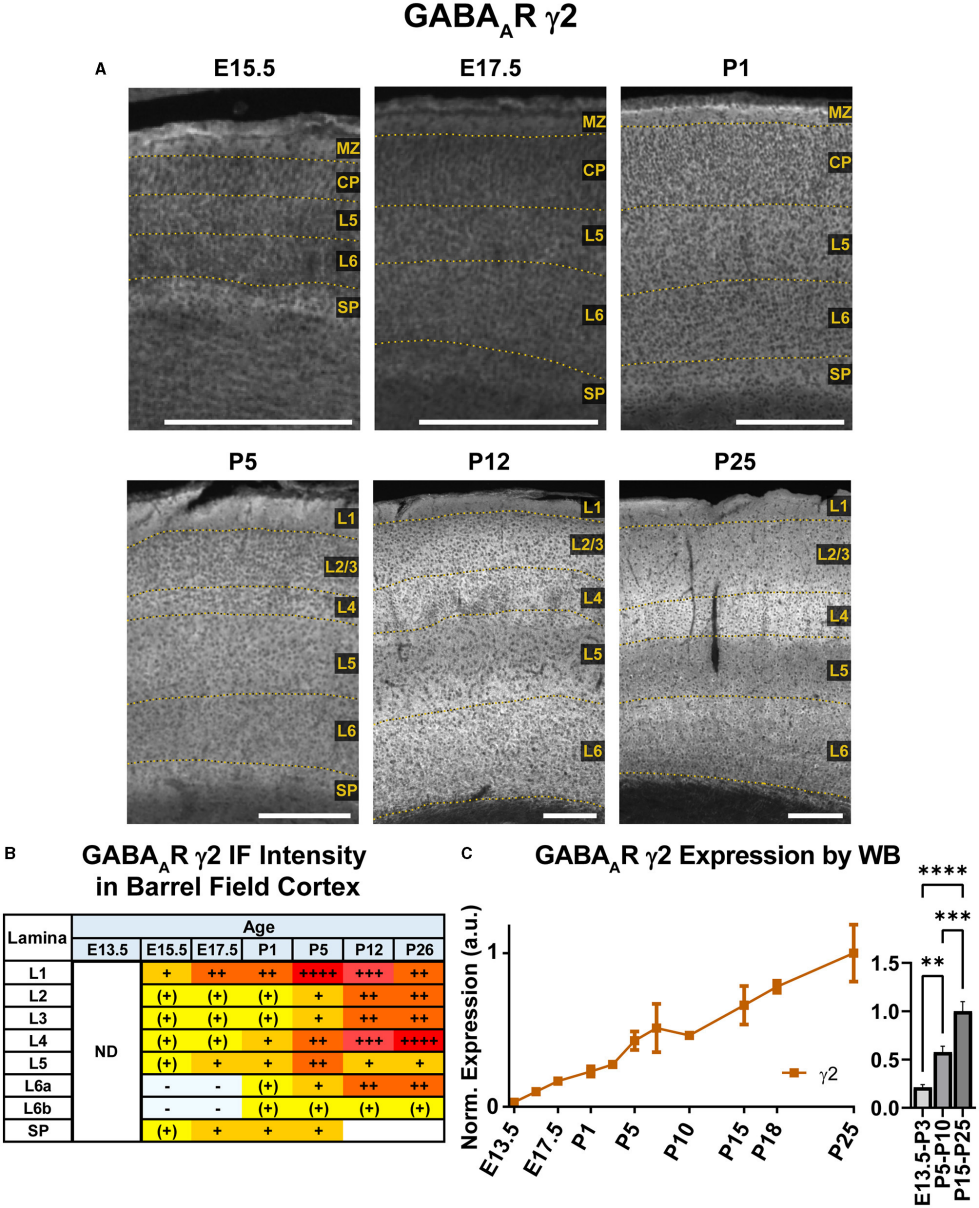
GABA_A_R γ2 expression in barrel field cortex from embryonic age to maturity. **(A)** Exemplar images of γ2 immunoreactivity in barrel field cortex across development. Of note, the sections shown here were stained at the same time, but L1 was damaged in the P12 sections. Scale bar = 250 μm. **(B)** Grading of immunofluorescence intensity across cortical lamina, where + + ++ is the maximal γ2 signal that could be detected in brain at P26. **(C)** Quantification of GABA_A_R γ2 protein in cortical samples by Western blot, with a timeline of all points on the left and binned stages tested for significance on the right. ^****^P < 0.0001, ^***^P < 0.001, ^**^P < 0.01 by one-way ANOVA with Šídák's multiple comparisons; n in mice/age: 3/E13.5, 3/E15.5, 3/E17.5, 6/P1, 3/P3, 3/P5, 3/P7, 3/P10, 4/P15, 4/P18, and 4/P25. L1–L6, layer 1–layer 6; MZ, marginal zone; SP, subplate; CP, cortical plate; ND, not determined; also refer to “Lamina in Grading Table and Figures” section in Methods.

Our WB results showed that γ2 expression begins early in development and exhibits a steady increase from E13.5 to P26 that was statistically significant (Figure 12; ^**^P < 0.01 E13.5-P3 vs. P5-P10, ^***^P < 0.0001 P15-P25 vs. P5-P10). Immunofluorescent stains showed a layer-specific pattern of γ2 expression (Figures 10, 12). As early as E15.5, we detected low levels of γ2 immunoreactivity in the MZ and subplate. At E17.5 and P1, γ2 was expressed throughout the CP, especially in L5. At P5, expression increased in all layers, but was highest in L4/L5 and was especially pronounced in the L4 barrels. At P12, γ2 expression was prominent in all layers, particularly high in L2-4 and L6. However, at the same time γ2 expression in L5 rose less significantly, so this layer was easily distinguishable from the higher expression in all other layers. Within L6, heightened expression was centered in L6a, but spread into L5c and top of L6b. These P12 patterns were preserved at P26.

### GABA_A_R β2

**Figure 13:**
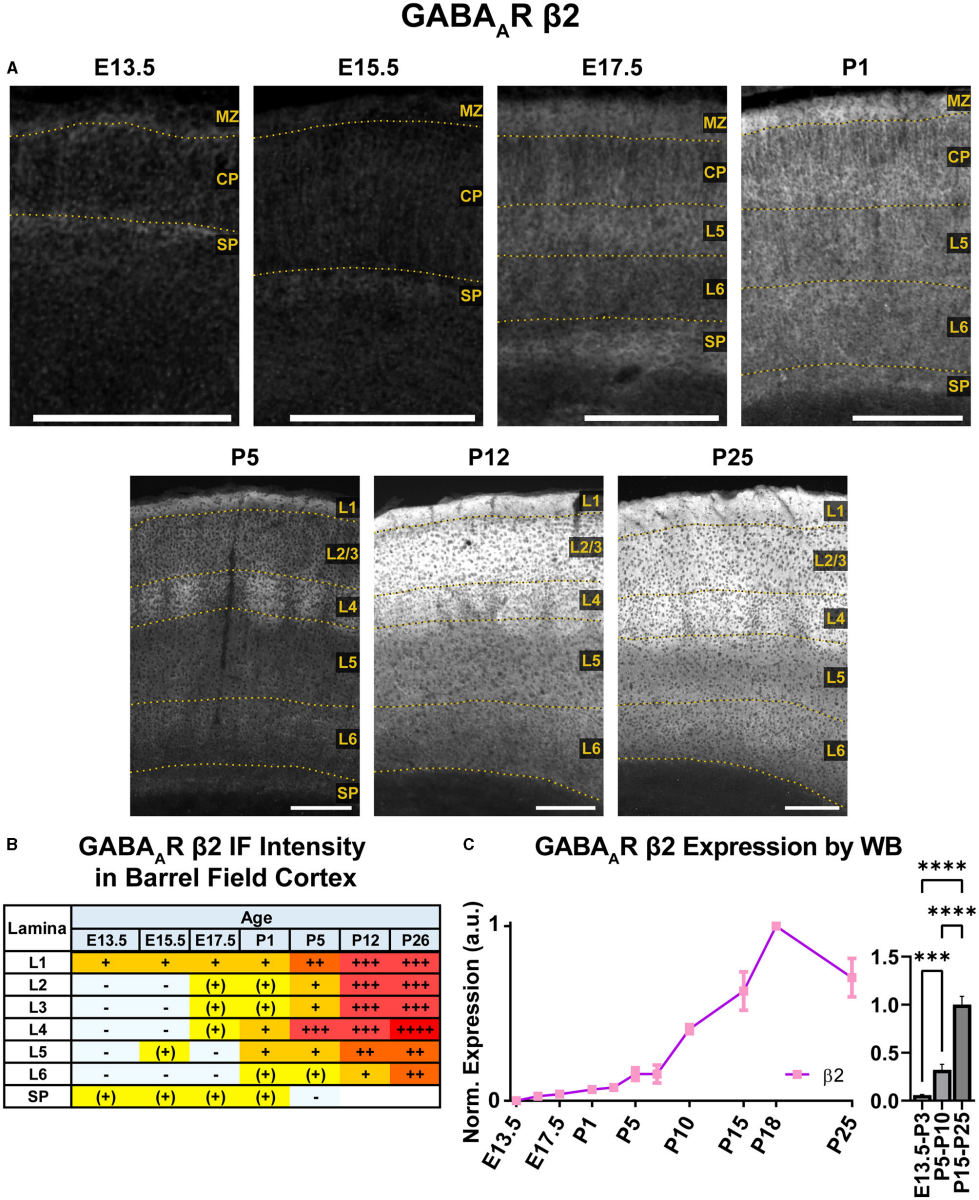
GABA_A_R β2 expression in barrel field cortex from embryonic age to maturity. **(A)** Exemplar images of β2 immunoreactivity in barrel field cortex across development. Scale bar = 250 μm. **(B)** Grading of immunofluorescence intensity across cortical lamina, where + + ++ is the maximal β2 signal that could be detected in brain at P26. **(C)** Quantification of GABA_A_R β2 protein in cortical samples by Western blot, with a timeline of all points on the left and binned stages tested for significance on the right. ^****^P < 0.0001, ^***^P < 0.001 by one-way ANOVA with Šídák's multiple comparisons; n in mice/age: 3/E13.5; 3/E15.5, 3/E17.5, 6/P1, 3/P3, 3/P5, 3/P7, 4/P10, 3/P15, 4/P18, and 3/P25. L1–L6, layer 1–layer 6; MZ, marginal zone; SP, subplate; CP, cortical plate; also refer to “Lamina in Grading Table and Figures” section in Methods.

WB immunoreactivity showed moderate β2 expression throughout the embryonic period that exhibited a steady increase to high levels in the postnatal period that was statistically significant (Figure 13; ^***^P < 0.001 E13.5-P3 vs. P5-P10, ^****^P < 0.0001 P5-P10 vs. P15-P25). In immunostained brain sections (Figures 10, 13), expression of β2 was seen as early as E13.5 in the MZ and subplate, followed by low levels of β2 in CP by E15.5/E17.5. This cortical expression was relatively featureless from E15.5 to P1, but showed a steady, progressive increase that was not as readily apparent in the WB data. A distinct subplate could be seen from E13.5–E17.5 but blended into L6 at P1 and later ages. Prominent expression in MZ/L1 became clear at E17.5 and was present at all ages.

Robust β2 expression appeared abruptly in L4 at P5, especially in barrel cortex. By P12, β2 expression expanded into L1–4, with lesser increases in L5/6. There was strong expression in L1–4 and somewhat lower levels in L5/6. By P26, L1–4 still had the highest expression, and weaker expression in L5.

### GABA_A_R β3

**Figure 14:**
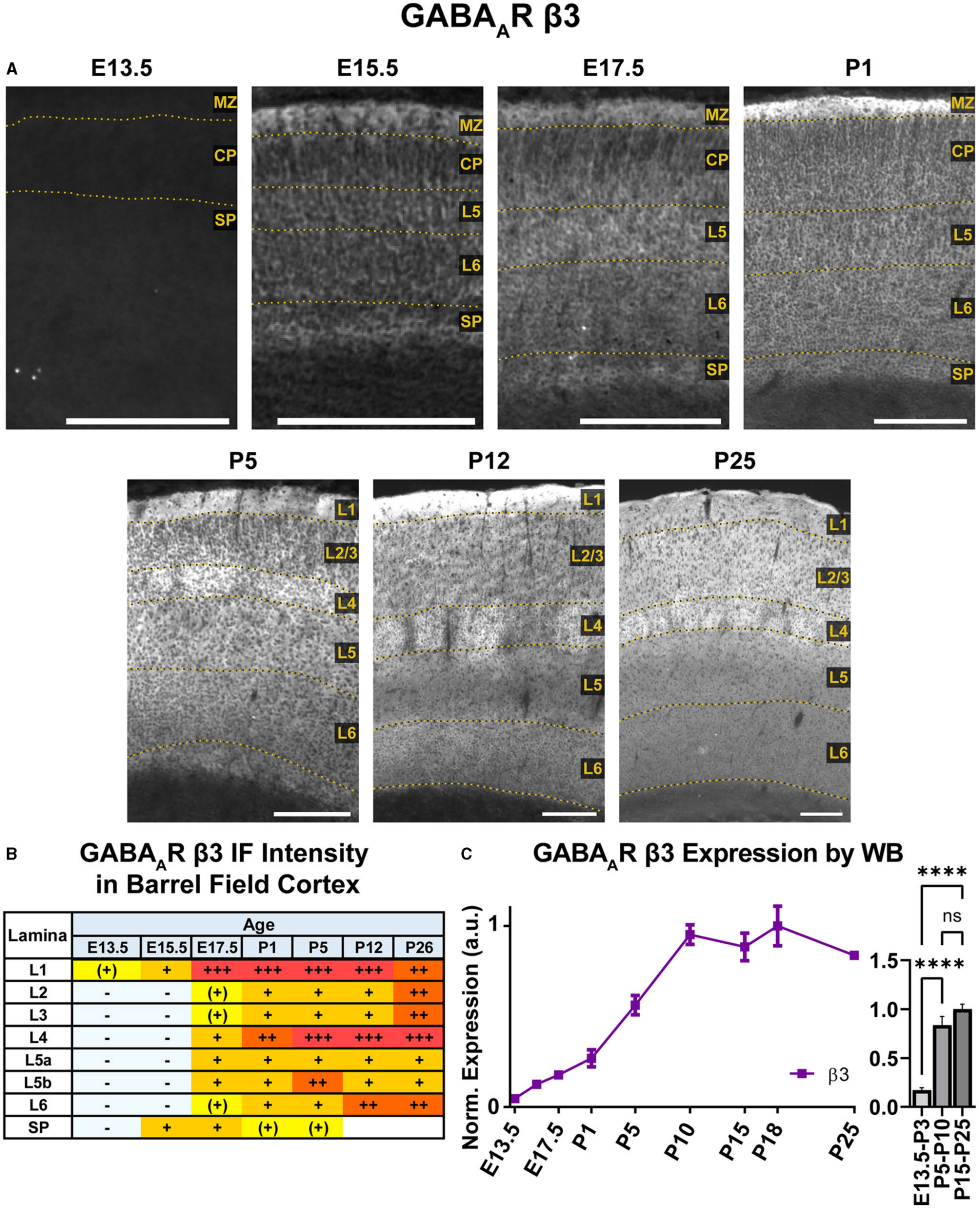
GABA_A_R β3 expression in barrel field cortex from embryonic age to maturity. **(A)** Exemplar images of β3 immunoreactivity in barrel field cortex across development. Scale bar = 250 μm. **(B)** Grading of immunofluorescence intensity across cortical lamina, where + + ++ is the maximal β3 signal that could be detected in brain at P26. **(C)** Quantification of GABA_A_R β3 protein in cortical samples by Western blot, with a timeline of all points on the left and binned stages tested for significance on the right. ^****^P < 0.0001 by one-way ANOVA with Šídák's multiple comparisons; n in mice/age: 4/E13.5, 4/E15.5, 4/E17.5, 4/P1, 4/P5, 4/P10, 4/P15, 4/P18, and 4/P25. L1–L6, layer 1–layer 6; MZ, marginal zone; SP, subplate; CP, cortical plate; also refer to “Lamina in Grading Table and Figures” section in Methods.

A steady general increase in cortical expression of β3 was apparent on WB, starting in the embryonic period and continuing until P10, when it reached a stable plateau of high expression that showed a statistically significant difference from embryonic/perinatal timepoints, but not across the postnatal period (Figure 14; ^****^P < 0.0001 P15-P25 and P5-P10 vs. E13.5-P3). In brain sections (Figures 10, 14), expression of β3 was first detectable at E15.5 in the MZ and subplate. Low expression was also visible in L5/L6 with L5 being slightly higher. While β3 expression is lower in L2–4, there are dendrites extending from L5 through L2/3 and ending in L1. Expression intensity increased from E15.5-P1, but the laminar pattern remained the same. By P5, β3 expression increased in the upper cortex, resulting in a relatively undifferentiated laminar pattern from L2 to the subplate. The one exception is in the barrel cortex, where the L4 barrels had notably higher expression than other layers and adjacent cortices. By P12, a clear, laminar pattern was again re-established throughout somatosensory and motor cortex due to a relatively weak β3 expression in L5, while L1 and L4 barrels had the highest expression. At P26, this general pattern persists, but L1 and L6 decrease in intensity, giving a pattern of high expression in L1-3, higher expression in L4/barrels, and moderate expression in L5-L6.

### KCC2

**Figure 15:**
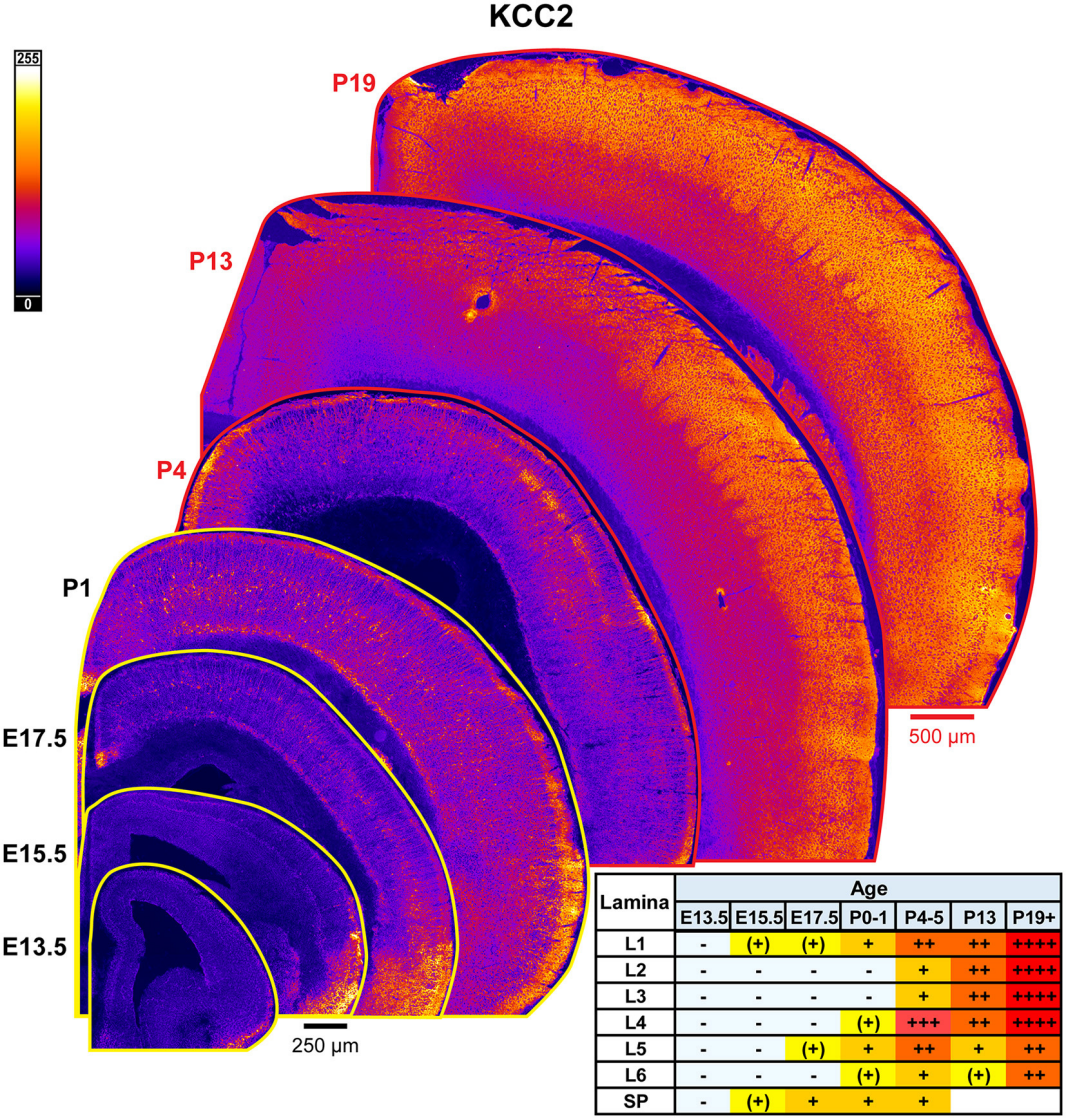
KCC2 expression in developing cortex. Coronal sections are overlaid from embryonic day E13.5 on the bottom left to postnatal day 19 on the top right, and concensus grading of immunofluorescence intensity is on bottom right. All sections are oriented from ventral bottom to dorsal top, with lateral cortex on the right. Separate scaling has been used for sections E13.5-P1 (black scale bar) and P4-P19 (red scale bar). Signal is represented using a heat map lookup table to highlight differences in regional expression. Heatmap intensity scaling is shown by the bar in top left. For grading of immunofluorescence intensity, + + ++ is the maximal KCC2 signal that could be detected in brain at P19+. L1–L6, layer 1–layer 6; SP, subplate; also refer to “Lamina in Grading Table and Figures” section in Methods.

The general pattern of KCC2 expression during rodent cortical development has been previously thoroughly characterized by WB and other techniques, showing low immunoreactivity during embryonic and perinatal life, and then a dramatic increase in the second to fourth postnatal weeks (Rivera et al., 1999; Stein et al., 2004; Dzhala et al., 2005; Uvarov et al., 2009; Takayama and Inoue, 2010; Kovács et al., 2014). Due to these prior studies, we only focused on investigating laminar and regional differences in KCC2 expression by IHC (Figure 15). We detected the earliest cortical KCC2 expression at E15.5 as faint immunoreactivity in the subplate and MZ. At E17.5-P1, KCC2 was robustly expressed in MZ and subplate. Additionally, individual neurons within the CP, particularly within L5, exhibited significant plasmalemmal KCC2 immunoreactivity that corresponds to GABAergic neuron-specific expression we previously reported (Zavalin et al., 2022). Early KCC2 expression within MZ also appeared to be interneuron-specific, since MZ is densely packed with migrating interneurons and similarly lost KCC2 immunoreactivity in the interneuron-specific KCC2 knockout. On the other hand, interneurons did not contribute to KCC2 expression within the subplate, which retained KCC2 immunoreactivity in the knockout (Zavalin et al., 2022). Therefore, with the exception of subplate-specific expression, KCC2 appears to be expressed exclusively by a subset of interneurons until P4-P5, at which point, we saw a marked increase in KCC2 expression within L4 and particularly the barrels, and low emerging expression in L5. At this point, we could also see numerous immunopositive dendrites ascending through L2/3. By P13, KCC2 was diffusely expressed throughout the cortical lamina and had higher expression in L1-4 than L5/6. Higher expression following a similar pattern was present after P18 (Figure 15).

## Discussion

### Synopsis of GABA_A_R subunit and KCC2 expression in developing cortex

In this study, we found unique spatial and temporal patterns of GABA_A_R subunits and KCC2 protein expression during cortical development. Generally, expression of α3 and α5 GABA_A_R subunits began very early, predominantly in L5/6 and subplate. On the other hand, α1, α2, α4, and δ, as well as KCC2, were primarily expressed at later developmental stages, most strongly in L4 and more superficial layers. In contrast, expression of β2, β3, and γ2 were spatially and temporally more ubiquitous than expression of α subunits but were similarly higher in certain laminae. β3 expression came online early and generally preceded expression of β2, although this difference was less distinct in the barrel field, *per se*.

### Rationale for our approach and comparison with other expression studies

While there have been a few studies reporting GABA_A_R subunit expression in perinatal cortex, they have been much more limited in scope than our work. For example, Fritschy et al. (1994) described α1 and α2 protein expression in somatosensory and visual cortex at P0, P4, and P20. In addition, Paysan et al. (1997) showed that α1, α2, α3, and α5 GABA_A_R expression at P7 in sensory cortex depended on perinatal thalamocortical input. More comprehensive evaluations of perinatal mRNA expression have been reported previously (Laurie et al., 1992), but lacked the layer-specific detail reported here. Other *in situ* hybridization studies have reported greater anatomic detail but did not include embryonic expression (Golshani et al., 1997; Fertuzinhos et al., 2014). More importantly, mRNA expression patterns may not match the subcellular distribution of functional GABA_A_Rs, such as mRNA expression in the somata but protein expression in the dendrites. Moreover, mRNA levels may not reflect quantitative differences in protein expression due to post-transcriptional and post-translational levels of control, such as mRNA editing, GABA_A_R internalization, and degradation. While our findings usually corroborate these previous publications on GABA_A_R expression (Laurie et al., 1992; Golshani et al., 1997; Hortnagl et al., 2013; Fertuzinhos et al., 2014) and KCC2 expression (Rivera et al., 1999; Stein et al., 2004; Dzhala et al., 2005; Uvarov et al., 2009; Takayama and Inoue, 2010; Markkanen et al., 2014; Zavalin et al., 2022), our work provides laminar resolution that was previously unknown.

### Lamina-specific expression

A summary of expression trajectories of multiple GABA_A_R subunit proteins within a single lamina is provided in Supplementary Figure 3. However, it is important to realize that differences in antibody affinity preclude any direct comparison of absolute protein quantity among the various subunits.

#### Layer 1/marginal zone

MZ/L1 is the site of multiple important development processes, including tangential migration of interneuron progenitors (Li et al., 2008; Bortone and Polleux, 2009; Bartolini et al., 2013), which rely on GABA_A_R-mediated excitation for motility (Inada et al., 2011). MZ is also populated by a transient neuronal population of Cajal-Retzius neurons, which develop exceptionally early and exhibit strong GABAergic input in the early period, playing a vital role in cortical circuit formation and organization (Kilb and Frotscher, 2016; Molnár et al., 2020). In our experiments, we found clear α2, α3, α5, β3, and γ2 GABA_A_R subunit expression in this layer by E15.5 in a generally diffuse pattern. At the time of birth, α1, α4, and δ were essentially absent in L1, but all other GABA_A_R subunits and KCC2 were clearly expressed.

#### Layers 2/3

L2/3 are the latest-maturing laminae in the inside-out sequence of cortical development. While α1, α2, α4, δ, and KCC2 expression was strong at maturity, these subunits were generally first clearly visible in L4 around P5 and then subsequently in more superficial layers by P12. This is concordant with the emergence of synaptic activity L2/3 and a critical period of receptive fields in L2/3 of barrel cortex (Maravall et al., 2004; Kobayashi et al., 2008). Prior to P5; α2, β2, β3, and γ2 were the predominant subunits expressed in L2/3. Additionally, α3 and α5 expression appeared to lie along the ascending dendrites of L4-6 neurons passing through L2/3. This interpretation is supported by previous work showing that α3 and α5 mRNA expression before P6-12 is predominantly in the lower and middle cortical layers, respectively (Laurie et al., 1992). From P5 onwards, the pattern of α3 expression became more diffuse and somewhat weaker, while α5 expression in barrel cortex virtually disappeared by P12.

#### Layer 4

L4 is the primary input layer for thalamocortical input, and dramatic changes in expression of GABA_A_R subunits and KCC2 occurred around the time when thalamocortical afferent fibers reach L4 at P4 and form defined barrels during P4-P8 (Inan and Crair, 2007). Prior to P5, GABA_A_R subunit expression in L4 was typically similar to L2/3. β3 and γ2 were consistently expressed in this area at all peri- and postnatal ages. At P5, there was an abrupt onset of strong α1, α2, α4, β2, δ, and KCC2 expression, while α3 and α5 expression was lost, and this pattern was also most prominent in barrel cortex. Expression of ɑ(1,2)β(2,3)γ2 pentamers could allow temporally precise GABAergic signaling for accurate sensory processing during and after the critical period, while extrasynaptic ɑ4βδ receptors may help provide local area regulation of multiple neurons with tonic inhibition.

#### Layers 5/6

L5/L6 are the earliest-forming cortical layers, and the site of early GABAergic “giant depolarizing potentials” that assist with circuit formation in the first postnatal week (Allene et al., 2008). Prior to P5, GABA_A_R subunits α3 and α5 were prominently expressed in L5/6 along with β2, β3, and γ2. KCC2 expression was also seen in L5 as early as E17.5, but was restricted to interneurons in the perinatal period, as previously shown with tissue from interneuron-specific KCC2 knockout mice (Zavalin et al., 2022). After P5, expression of α3 and α5 remained robust, but gained a sublaminar pattern with slightly stronger expression in the superficial L5 (L5a), and the border between L5 and L6 (L5c/L6a). At P12 there were moderate levels of α1 and γ2 expression in L1-4 and L6, but not L5. Finally, while α2, α4, δ, and KCC2 expression began in L5/6 by P12, it remained relatively weak compared to more superficial layers.

#### Subplate

Like the MZ, subplate hosts a population of transient, early-developing neurons that assist cortical formation by regulating processes like thalamocortical axon pathfinding and radial migration of neurons. Subplate neurons form a layer beneath the CP, with a distinct cell-sparse zone between L6 and the subplate from E15.5 until at least P2 (Torres-Reveron and Friedlander, 2007). Some of these subplate neurons are GABAergic, and prominent GABAergic currents can be evoked in subplate neurons (Unichenko et al., 2015; Ohtaka-Maruyama, 2020). Previous work has shown that after P2-4, a distinct layer of subplate neurons is lost, and these cells become intermixed with the lower portions of L6 (Kast and Levitt, 2019).

We found strong expression of GABA_A_R subunits α3, α5, and β2 that began as early as E13.5/E15.5; followed by β3, γ2, and KCC2 expression by E15.5/E17.5; and then low α1 expression by E17.5/P1. In addition, there was faint, primarily somatic δ expression in some subplate neurons as early as P1, as previously reported by Qu et al. (2016). This somatic pattern was most evident at P5 and persisted with fewer cells at P12 in the region where subplate was found earlier, and then was essentially gone by P26.

Previous work has shown *Gabra5* mRNA in the perinatal subplate (Golshani et al., 1997), and we found a consistent band of α5 expression restricted to the subplate. In contrast to other subunits, this expression persisted even into adulthood as a distinct, thin band in the post-subplate region/L6b. In contrast, α3 and β3 had a broader band of expression that included both the subplate, as well as the cell-poor region between the subplate and CP (referred to as “L6b” by Catalano et al., 1991). Unlike *Gabra5, Gabra3* mRNA is not known to be expressed in subplate at this age, and some α3 protein expression may be due to afferent/efferent fibers passing through this area, such as thalamocortical input and corticothalamic output. In particular, growing thalamocortical afferents that eventually project to L4 make contact with subplate neurons at E16-E19 (Catalano et al., 1991; Inan and Crair, 2007).

### Other notable expression patterns

#### Subcortical α3+ fiber tracks

While GABA_A_R expression is not typically found in the subcortical white matter of a mature brain, we found expression of α3 in fibers of the intermediate zone and internal capsule in the embryonic brain between E15.5 to P1, which was most evident at E17.5. This expression was no longer present by P5 and was also not seen in tissue from E17.5 *Gabra3*-knockout mice. Embryonic thalamus expresses α3 (Laurie et al., 1992, also visible in Figure 7A), and α3 expression can be seen extending past the striatum and into the subcortical region with a pattern suggestive of thalamocortical fibers (Agmon et al., 1993; Bicknese et al., 1994; Abe et al., 2015). Conversely, L5/6 show robust α3 expression starting in the embryonic period, and the fibers may also mark corticothalamic tracks. Future work will need to be done to more specifically identify the source of this α3 expression and its developmental significance.

#### Radial glia

α2 GABA_A_R protein expression was found in the superficial subcortical tissue, with radially oriented projections extending through the overlying CP in the embryonic and perinatal periods. Our results show that this α2 GABA_A_R protein expression overlaps with a subset of RC2-labeled radial glia in dorsal cortex. Previous work has shown that both mature glia and their precursors express functional GABA_A_Rs (Wang et al., 2005; Muth-Kohne et al., 2010; Renzel et al., 2013). Bergmann glia specifically express *Gabra2* mRNA (Riquelme et al., 2002), and possibly α1, β1, β3, and γ1 (Bovolin et al., 1992). Laurie et al. (1992) also reported mRNA expression of α2 in the lower intermediate zone by E17. Thus, it is possible that our results reflect presence of α2-containing GABA_A_Rs in the radial glia. However, radial glial processes in embryonic brain are much more widely distributed than the pattern of α2 expression reported here. There were RC2-positive fibers extending centrifugally from both pallial and subpallial ventricular zones, while α2+ fibers lacked this range and appeared to arise from the intermediate zone. Therefore, the identity of these fibers is not entirely clear. It is conceivable that these fibers represent radial processes of a subset of radial glia, but it is just as likely that they are expressed in some other closely associated processes instead. Additionally, it is unclear whether these are functional GABA_A_R pentamers, since we did not see this pattern with other GABA_A_R proteins.

#### Somata

Immunolabelling for the δ GABA_A_R subunit or KCC2 identified distinct somata in cortex. In the case of KCC2, we were able to corroborate that this represented early KCC2 expression in interneurons (Zavalin et al., 2022). The δ+ somata were most prominent at P12 and P26, but the identity of these cells is unclear. A similar pattern has been reported in hippocampus by other groups, which found discrete α1, β2, and δ co-expressing cell bodies that correspond with parvalbumin interneurons (Peng et al., 2004; Milenkovic et al., 2013). We also found intensely labeled α1+ and β2+ somata in hippocampus from P5-P26 (data not shown), but not in the cortex from the same sections. Therefore, cortical δ+ somata may likewise represent interneurons, but it is unclear which GABA_A_R subunits partner with δ in the cortex, although α4β2δ is the subunit combination most commonly found in brain.

### Developmental significance of subunit expression patterns

The developmental significance of the expression patterns discussed here is unclear without knowing how those changes will affect GABAergic signaling. Fortunately, there is now a large body of work characterizing the pharmacodynamic properties and subcellular location of different GABA_A_R subunit-combination (Chuang and Reddy, 2018; Engin et al., 2018).

#### γ2-containing GABA_A_Rs

GABA_A_Rs containing the γ2 subunit are the primary mediators of synaptic responses, since they are often found within the synapse and tend to produce large, rapidly activating and deactivating currents. However, they require relatively high [GABA] (≈ 10–15 μM) for full activation and also desensitize rapidly. We found that ubiquitous γ2 expression begins early in development and ramps up to even higher levels as the brain matures, suggesting that γ2-containing GABA_A_Rs constitute a sizeable portion of cortical GABA_A_Rs at all ages. Moreover, some of the α subunits that typically combine with γ2, which include α1, α2, α3, and α5 in adult brain, are also present at early ages. However, expression of each of these α subunits significantly varies by developmental stage and lamina, imparting different properties to γ2-containing GABA_A_Rs in development and adulthood.

#### α3 and α5-containing GABA_A_Rs

Among the α subunits, α3 and α5 are expressed particularly early in development in multiple cortical laminae, including the MZ, L5, and subplate. Unlike in adult brain, these subunits often had spatially and temporally overlapping patterns of expression in the developing cortex. GABA_A_Rs containing either of these subunits tend to have prolonged decay times and slow desensitization. Concordantly, GABAergic currents during embryonic and perinatal period generally have slow phasic or tonic kinetic properties (Le Magueresse and Monyer, 2013; Warm et al., 2022). However, α3- and α5-containing GABA_A_Rs have notably different sensitivities to GABA (Picton and Fisher, 2007; Rula et al., 2008; Lagrange et al., 2018). For example, α5β3γ2 GABA_A_Rs are sensitive to low [GABA] (< 5–10 μM) and often localize to the extrasynaptic space, allowing them to convey much of the tonic inhibition in some adult brain regions (Caraiscos et al., 2004; Lagrange et al., 2018). In contrast, α3-containing GABA_A_Rs require very high [GABA] (EC50 30–100 μM) and relatively prolonged or repetitive exposure to GABA for full activation (Rula et al., 2008). Thus, a mixed population of α3βγ2 and α5βγ2 GABA_A_Rs would provide a pool of highly sensitive α5-containing GABA_A_Rs to respond to low levels of GABA and another population of α3-containing GABA_A_Rs tuned to detect repetitive exposure to high [GABA]. The ability of α3-containing GABA_A_Rs to detect coincident stimulation may be particularly useful in a developmental context, where growth cone stabilization, synaptogenesis, and other developmental processes depend on repetitive GABA_A_R activation. Indeed, embryonic α3 expression is required for the formation of certain axo-axonic synapses in the retina (Sinha et al., 2021). Interestingly, α3 expression may provide an additional, temporally dependent regulation of GABAergic signaling. *Gabra3* is subject to RNA editing during later stages of development (50% edited at P2-5, 90% edited at P7-9). This process converts an isoleucine to methionine in the third transmembrane domain, leading to reduced GABA potency, reduced surface expression, and faster decay (Rula et al., 2008; Daniel et al., 2011), which likely fine-tunes the ability to sum up repetitive stimuli.

On the other hand, α5-containing GABA_A_Rs may provide GABAergic depolarization when low [GABA] is present. Sebe et al. (2010) found significant α5-mediated tonic current in cortical L5 neurons uniquely at early postnatal timepoints, which excited a minority and inhibited the majority of neurons, but whether similar tonic currents occur in other areas remains to be determined. While α5-containing GABA_A_Rs tend to be extrasynaptic in adult brain, they have also been found at/near synapses in developing neurons and mediate signals that assist neuronal development (Serwanski et al., 2006; Brady and Jacob, 2015; Hernandez et al., 2019). Previous work has shown that α5-mediated currents promote dendrite and spine development *in vitro* (Giusi et al., 2009; Brady and Jacob, 2015), as well as migration and dendrite development of adult-born granule cells (Deprez et al., 2016; Lodge et al., 2021).

#### α1 and α2-containing GABA_A_Rs

In contrast to α3 and α5, α1 and α2-containing GABA_A_Rs have kinetic properties that are tailored toward phasic signaling associated with mature synaptic signals. The robust upregulation of these two subunits is coincident with a period of maximal synaptic formation during the second and early third postnatal week in mice (Bosman et al., 2002; Kobayashi et al., 2008; Okaty et al., 2009; Lazarus and Huang, 2011; Le Magueresse and Monyer, 2013; Yang et al., 2014).

α1βγ2 is by far the most abundant GABA_A_R subunit combination found in adult brain and conveys the majority of synaptic inhibition. α1βγ2 GABA_A_Rs activate very quickly and have moderate rates of deactivation that allow them to convert sub-millisecond GABA transients into currents lasting tens of milliseconds or more. However, these GABA_A_Rs also desensitize quickly and extensively. Thus, synaptic activity conveyed by α1βγ2 GABA_A_R is able to respond to sparse synaptic activity with high temporal precision but is also relatively insensitive to high frequency input (Bianchi et al., 2007; Lagrange et al., 2018). Indeed, electrophysiological experiments in L2/3 show a developmental transition of GABAergic synaptic currents toward a fast activation/fast deactivation profile, which is coincident with a decrease of the α3-mediated component and increase in the α1-mediated component (Bosman et al., 2002; Kobayashi et al., 2008).

α2βγ2 have similar activation, deactivation, and desensitization to α1βγ2, but somewhat lower GABA potency and more rapid recovery during frequent stimulation (Picton and Fisher, 2007). While α1βγ2 GABA_A_Rs are prevalent at the majority of GABAergic synapses (Chuang and Reddy, 2018; Engin et al., 2018), α2βγ2 GABA_A_Rs appear to be the dominant combination at specific synapses, such as synapses made by parvalbumin+ chandelier interneurons on the axon initial segment and on somatic synapses made by non-parvalbumin basket interneurons, such as cholecystokinin+ interneurons (Nusser et al., 1996; Nyíri et al., 2001; Klausberger et al., 2002).

#### δ-containing GABA_A_Rs

In contrast to synaptic γ2-containing GABA_A_Rs, α5βγ2 and δ-containing GABA_A_Rs are mediators of tonic GABAergic signaling, partly due to their preferential localization outside of the synapse. The δ subunit tends to partner with α4 *in vivo* to create α4βδ pentamers (Engin et al., 2018; Lagrange et al., 2018), and we found that α4 and δ followed a similar pattern of expression that was quite sparse until the second postnatal week. α4βδ GABA_A_Rs are the primary mediators of tonic inhibition in response to low levels of extrasynaptic [GABA] found in cortex, thalamus, and hippocampus (usually ≈ 1 μM or even less), activating slowly but maintaining a prolonged tonic current due to slow deactivation and low desensitization (Lagrange et al., 2018). Since α4βδ GABA_A_Rs are maximally activated at low [GABA], they can only discriminate a relatively narrow range of extrasynaptic [GABA] concentrations (≤ 1–5 μM). α5β3γ2 GABA_A_Rs are less sensitive to low [GABA], with EC50s between those of α4βδ and α1βγ2 GABA_A_Rs (Lagrange et al., 2018). While α4 and α5 subunits are often expressed in different brain regions, there are some areas of overlap (Hortnagl et al., 2013). In these cases, expression of both α4βδ and α5β3γ2 GABA_A_Rs imparts the ability to fine-tune network activity to a wider range of [GABA] (Scimemi et al., 2005).

However, α4 and α5 subunits often have non-overlapping patterns of expression. As mentioned previously, α5-containing GABA_A_Rs convey most of the tonic signaling in early life, since α4 and δ subunit expression only appears around P5. Even in the adult cortex, where both subunits are expressed, α5 is expressed highly in L5/6, α4 expression is higher in superficial layers. Neurons throughout the cortical column have been shown to exhibit tonic currents, but subunit composition of GABA_A_Rs mediating these currents differs by layer (Yamada et al., 2007; Jang et al., 2013). Therefore, while tonic currents in L5 have a prominent α5-mediated component (Yamada et al., 2007), our findings indicate that tonic currents in L4 and superficial layers may have stronger α4 and δ-mediated components.

A small percentage of the α1 subunit can also be found in α1βδ GABA_A_Rs, which are predominantly expressed on interneurons (Glykys et al., 2007) that could correspond with the immunolabeled somata we observed for both α1 and δ subunits. These pentamers have GABA potency that is similar to α5β3γ2, but also have very fast rates of activation and deactivation, as well as much less desensitization than any other GABA_A_R subunit combinations studied so far (Bianchi et al., 2002; Lagrange et al., 2018). Their extremely fast deactivation would make them poorly suited to respond to low frequency phasic stimulation. Their minimal desensitization is conducive to tonic inhibition, but kinetic properties make this subunit combination able to respond near-instantaneously to abrupt changes in extrasynaptic GABA. These properties are expected to produce extrasynaptic responses with extremely high temporal precision to presynaptic input, but little overall charge transfer during single events. α1βδ GABA_A_Rs are well-suited to respond to prolonged, repetitive synaptic input with high temporal fidelity.

#### KCC2

KCC2 has a strong influence on development through its effect in transitioning GABAergic signaling to mediate inhibition during later stages of development. In agreement with this function, we saw late KCC2 expression in cortex with exception of interneurons, where KCC2 may play an important developmental role (Cuzon et al., 2006; Bortone and Polleux, 2009; Inada et al., 2011; Inamura et al., 2012; Zavalin et al., 2022). While KCC2 expression is a significant factor in heralding a transition to inhibitory GABAergic responses, extensive regulation by kinases further restricts KCC2 activity to late stages of development (Fukuda, 2020; Virtanen et al., 2020, 2021), and additional factors including extracellular matrix proteins influence the polarity of GABAergic responses (Delpire and Staley, 2014; Glykys et al., 2014; Rahmati et al., 2021). Additionally, KCC2 has transport-independent functions that affect dendritic spine formation, apoptosis, and other developmental processes (Llano et al., 2020).

We did not distinguish the two isoforms of KCC2, KCC2a, and KCC2b, which both act as transporters, but have structural differences and significantly vary in temporal and spatial expression (Uvarov et al., 2007, 2009; Markkanen et al., 2014, 2017).

### Additional considerations

Our data from P26 mice is generally quite consistent with the published literature. However, we cannot rule out the possibility that tissue from later ages might have revealed greater changes in expression intensity, such as a drop in α2 or α5 expression. We did not measure expression of β1, γ1, and γ3, as we were unable to find a suitably specific antibody for use in WB and IHC experiments.

Unlike other subunits, it is less clear how much β subunit composition affects GABA_A_Rs properties, and the significance of asynchronous β2 and β3 in cortical signaling is unclear. However, there are some subtle differences between β2 and β3 containing GABA_A_Rs, such as their sensitivity to some general anesthetics (Zeller et al., 2007), and α4β3δ are less sensitive to low GABA than α4β2δ GABA_A_Rs (Lagrange et al., 2018). In general, α5 tends to partner with β3 and γ2 *in vivo*, so early expression of β3 and γ2 may be conducive to forming α5β3γ2 GABA_A_Rs (Mckernan and Whiting, 1996).

The isoform specific GABA_A_R responses are only one factor mediating GABAergic signaling in the developing brain. The concentration and kinetics of GABA play an equally important role that may vary from very brief synaptic transients to longer bursts of synaptic input, slower transients of intermediate GABA concentrations, and even steady state levels of low GABA (Brickley and Mody, 2012).

This work evaluated the expression of individual GABA_A_R subunits, and thus is not entirely informative of subunit combinations that form functional GABA_A_Rs. Though certain frequently-occurring combinations can be predicted from our expression, we cannot address the effects of mixed GABA_A_R α subunit combinations that are expressed *in vivo* (Sun et al., 2023). For example, it is currently unknown whether α3/α5β3γ2 would have high sensitivity to GABA like α5β3γ2 GABA_A_Rs, or low like α3β3γ2 GABA_A_Rs. Furthermore, a number of endogenous GABA_A_R modulators, such as neurosteroids and endozepines, are also developmentally regulated and regulate GABA_A_R function (Brown et al., 2016; Tonon et al., 2020). Finally, GABA_A_R-mediated signals can differ not only by lamina and developmental stage, but by specific neuronal types, circuits, and synapses. Therefore, while our experiments delineate certain trends in cortical GABA_A_R composition during development, physiology studies that focus on certain neuronal types and synapses may find exceptions and specializations that do not follow these trends.

## Data availability statement

The datasets presented in this article are not readily available because imaging and expression data has not been systematically organized for shared use and will not be stored indefinitely. Requests to access the datasets should be directed to AL, andre.h.lagrange@vumc.org.

## Ethics statement

Ethical approval was not required for the studies on humans in accordance with the local legislation and institutional requirements because only commercially available established cell lines were used. The animal study was approved by the Vanderbilt University Institutional Animal Care and Use Committee. The study was conducted in accordance with the local legislation and institutional requirements.

## Author contributions

KZ: Formal analysis, Investigation, Methodology, Visualization, Writing – original draft, Writing – review & editing. AH: Formal analysis, Investigation, Methodology, Visualization, Writing – review & editing. YZ: Investigation, Methodology, Writing – review & editing. ZK: Investigation, Writing – review & editing. AL: Conceptualization, Formal analysis, Funding acquisition, Investigation, Methodology, Project administration, Supervision, Visualization, Writing – review & editing.
