# Supplementary material for: Region and layer-specific expression of GABAA receptor isoforms and KCC2 in developing cortex
Source: Front Cell Neurosci. 2024 Jun 4;18:1390742. doi: 10.3389/fncel.2024.1390742 (PMC11184147; doi:10.3389/fncel.2024.1390742)
Supplement: Supplementary file 1 [file Data_Sheet_1.PDF]

Primary Antibodies						
**Antibody**	**Host**	**Vendor Name**	**Catalog Number**	**IHC Dilution**	**WB Dilution**	**RRID**
α1 GABA-A receptor	Rabbit	Millipore	06-868	1:500	1:500	AB_310272
α2 GABA-A receptor	Rabbit	Abcam	ab72445	1:250	1:500	AB_1268929
α3 GABA-A receptor	Rabbit	Alomone	AGA-003	1:500	1:1000	AB_2039866
α4 GABA-A receptor	Rabbit	Novus	NB300-194	1:500	1:500	AB_2109118
α5 GABA-A receptor	Rabbit	Millipore	AB9678	1:250	1:500	AB_570435
β2 GABA-A receptor	Rabbit	Millipore	AB5561	1:250	1:500	AB_177524
β3 GABA-A receptor	Rabbit	Novus	NB300-199	1:250	1:500	AB_2232287
δ GABA-A receptor	Rabbit	R&D Systems	PPS090	1:250	1:500	AB_2109428
γ2 GABA-A receptor	Rabbit	Synaptic Systems	224-003	1:250	1:2000	AB_2263066
RC2	Mouse	DSHB	RC2	1:50	n/a	AB_531887
KCC2	Rabbit	EMD-Millipore/Upstate	07-432	1:250	n/a	AB_310611
E6AP	Mouse	Sigma	E8655	n/a	1:1000	AB_261956
Actin	Mouse	Millipore	MAB1501	n/a	1:20000	AB_2223041
**Secondary Antibodies**						
**Antibody Name**	**Host**	**Vendor Name**	**Catalog Number**	**IHC Dilution**	**WB Dilution**	**RRID**
anti-Rabbit Cy3	Donkey	Jackson ImmunoResearch	711-165-152	1:1000	n/a	AB_2307443
anti-Mouse Cy3	Donkey	Jackson ImmunoResearch	715-165-150	1:500	n/a	AB_2340813
anti-Mouse Alexa 647	Donkey	Jackson ImmunoResearch	715-605-150	1:250	n/a	AB_2340862
anti-rabbit IRDye680RD	Goat	Li-COR	926-68071	n/a	1:5000-1:10000	AB_10956166
anti-mouse IRDye800CW	Goat	Li-COR	926-32210	n/a	1:5000-1:10000	AB_621842
anti-rabbit IRDye800CW	Goat	Li-COR	926-32211	n/a	1:5000-1:10000	AB_621843

Supplementary Table 1. List of antibodies used.

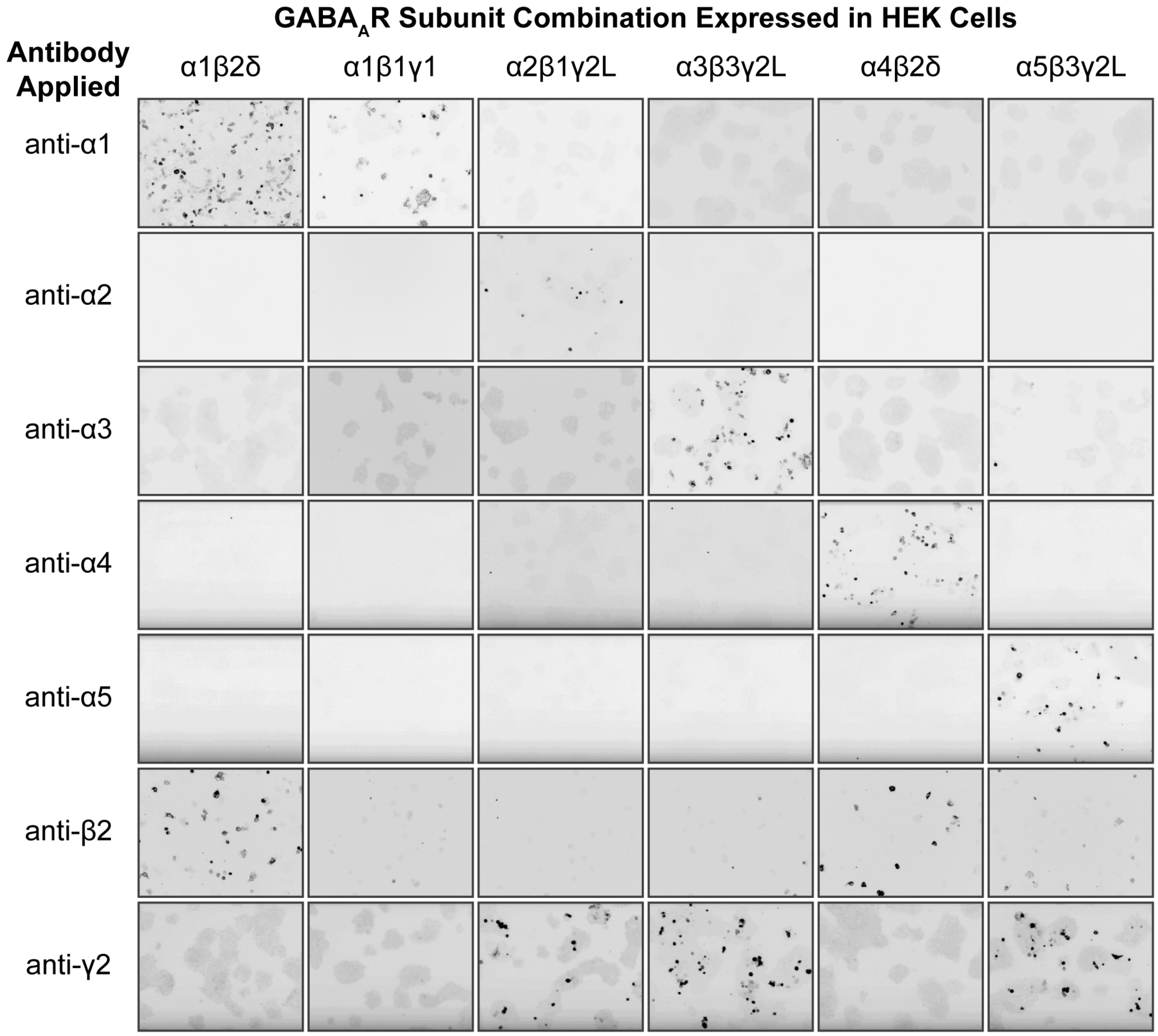

Supplementary Figure 1. Confirming specificity of antibodies to target $GABA_AR$ subunits in HEK cells. $GABA_AR$ subunit combinations expressed in each culture are listed in the top row, while the antibodies are in the left column. Note that the black immunolabeling signal is only present when antibodies are applied to culture expressing the target subunits. We also confirmed the specificity of anti-β3 and anti-δ antibodies, which are not shown in this figure.

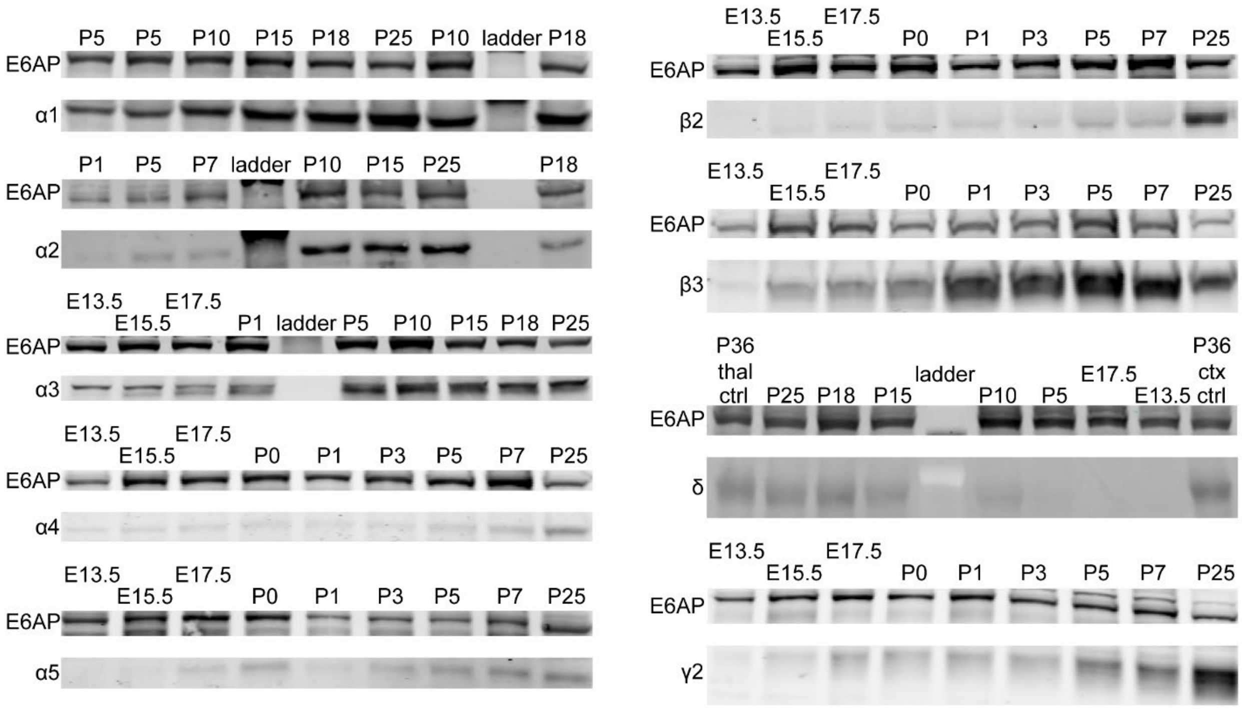

Supplementary Figure 2. Sample Western blot bands. Exemplar Western blot bands that were used to quantify levels of $GABA_AR$ α1-5, β2-3, δ, and γ2 are shown with normalization control E6AP. Age at which cortical samples were collected is indicated above each lane. Some of the blots do not include all ages assayed in this study, which were run on separate blots (not shown) with overlapping timepoints for control. P36 thalamus control and P36 cortex control denote P36 thalamic and cortical sample reference controls, respectively. Approximate molecular weights: E6AP 100 kDa, α1 52 kDa, α2 51 kDa, α3 55 kDa, α4 61 kDa, α5 52 kDa, β2 59 kDa, β3 54 kDa, δ 51 kDa, γ2 ~45 kDa.

Lamina-Specific Expression of Multiple GABA$_A$R Subunits and KCC2

LAYER 1/MARGINAL ZONE

		Age						
		E13.5	E15.5	E17.5	P1	P5	P12	P26
α1	L1	ND	-	(+)	(+)	+	++++	++++
α2	L1	+	+	+	++	++	++	+
α3	L1	+	++	++++	++++	+++	++	++
α4	L1	-	-	-	-	-	+	+++
α5	L1	ND	(+)	++	+	+	++	+
δ	L1	ND	(+)	-	(+)	-	+	+
γ2	L1	ND	+	++	++	++++	++++	++
β2	L1	+	+	+	+	++	+++	+++
β3	L1	(+)	+	+++	+++	+++	+++	++
KCC2	L1	-	(+)	(+)	+	++	++	++++
		Marginal Zone				Layer 1		

LAYER 2

		Age						
		E13.5	E15.5	E17.5	P1	P5	P12	P26
α1	L2	ND	-	-	(+)	(+)	++	+++
α2	L2	-	(+)	+	+	(+)	++	++
α3	L2	(+)	(+)	+	++	+	++	+
α4	L2	-	-	-	-	(+)	+	++
α5	L2	ND	-	+	(+)	++	+	+
δ	L2	ND	(+)	(+)	-	-	+	+
γ2	L2	ND	(+)	(+)	(+)	+	++	++
β2	L2	-	-	(+)	(+)	+	+++	+++
β3	L2	-	-	(+)	+	+	+	++
KCC2	L2	-	-	-	-	+	++	++++
		Cortical Plate				Layer 2		

LAYER 3

		Age						
		E13.5	E15.5	E17.5	P1	P5	P12	P26
α1	L3	ND	-	-	(+)	+	+++	++++
α2	L3	-	(+)	(+)	(+)	(+)	++	++
α3	L3	(+)	(+)	+	++	+	++	+
α4	L3	-	-	-	-	(+)	++	++
α5	L3	ND	-	++	+	++	+	+
δ	L3	ND	(+)	(+)	-	-	+	+
γ2	L3	ND	(+)	(+)	(+)	+	++	++
β2	L3	-	-	(+)	(+)	+	+++	+++
β3	L3	-	-	(+)	+	+	+	++
KCC2	L3	-	-	-	-	+	++	++++
		Cortical Plate				Layer 3		

LAYER 4

		Age						
		E13.5	E15.5	E17.5	P1	P5	P12	P26
α1	L4	ND	-	(+)	+	++	+++	++++
α2	L4	-	(+)	(+)	+	++	+++	++
α3	L4	(+)	(+)	++	+++	+	(+)	(+)
α4	L4	-	-	-	(+)	++	++++	+++
α5	L4	ND	-	++	+	+	(+)	(+)
δ	L4	ND	(+)	(+)	-	+	+++	+++
γ2	L4	ND	(+)	(+)	+	++	+++	++++
β2	L4	-	-	(+)	+	+++	+++	++++
β3	L4	-	-	+	++	+++	+++	+++
KCC2	L4	-	-	-	(+)	+++	++	++++
		Bottom of Cortical Plate				Layer 4		

LAYER 5

		Age						
		E13.5	E15.5	E17.5	P1	P5	P12	P26
α1	L5	ND	-	(+)	(+)	(+)	+	+
α2	L5	-	-	-	(+)	(+)	+	+
α3	L5a	(+)	+	+++	++++	+++	++	+
α3	L5b	(+)	+	+++	++++	+++	+	+
α4	L5	-	-	-	-	(+)	+	+
α5	L5a	ND	-	+++	+++	++	++	+
α5	L5b	ND	-	++	+	+	+	(+)
α5	L5c	ND	-	++	+	++	++	+
δ	L5	ND	(+)	(+)	(+)	-	+	+
γ2	L5	ND	(+)	+	+	++	+	+
β2	L5	-	(+)	-	+	+	++	++
β3	L5a	-	-	+	+	+	+	+
β3	L5b	-	-	+	+	++	+	+
KCC2	L5	-	-	(+)	+	++	+	++

LAYER 6

		Age						
		E13.5	E15.5	E17.5	P1	P5	P12	P26
α1	L6a	ND	-	(+)	(+)	(+)	++	++
α1	L6b	ND	-	-	(+)	(+)	+	++
α2	L6	-	(+)	+	+	(+)	++	++
α3	L6	-	(+)	+++	+++	++	+	+
α4	L6	-	-	-	(+)	(+)	+	+
α5	L6	ND	-	++	+	+	+	+
δ	L6	ND	(+)	-	(+)	-	(+)	(+)
γ2	L6a	ND	-	-	(+)	+	++	++
γ2	L6b	ND	-	-	(+)	(+)	(+)	(+)
β2	L6	-	-	-	(+)	(+)	+	++
β3	L6	-	-	(+)	+	+	++	++
KCC2	L6	-	-	-	(+)	+	(+)	++

SUBPLATE

		Age				
		E13.5	E15.5	E17.5	P1	P5
α1	SP	ND	-	(+)	(+)	(+)
α2	SP	-	-	-	-	-
α3	SP	+	+	+++	+++	+++
α4	SP	-	-	-	-	-
α5	SP	ND	+++	++	+	++
δ	SP	ND	-	-	-	-
γ2	SP	ND	(+)	+	+	+
β2	SP	(+)	(+)	(+)	(+)	-
β3	SP	-	+	+	(+)	(+)
KCC2	SP	-	(+)	+	+	+

Supplementary Figure 3. Semi-quantitative grading of GABA$_A$R α1-5, β2-3, δ, γ2, and KCC2 expression organized by lamina. Each table represents expression within a single lamina from E13.5 to P26, going left to right across columns. The transition from transient developmental lamina to cortical layers is distinguished at the bottom of the tables. Each row represents a different GABA$_A$R subunit or KCC2, and sublaminar distinctions, which are color-coded and labeled on the left of each table. Note that comparing absolute levels of expression between proteins should be avoided due to different antibody affinities and normalized grading for each of the proteins, e.g. a "++++" grading for different proteins indicates relative peaks in expression of each protein rather than the same amount of the two proteins. Abbreviations: L1-L6, layer 1-layer 6; SP, subplate; ND, not determined; also refer to "Lamina in Grading Table and Figures" section in Methods.
